# Parallel evolution of salinity tolerance in *Arabidopsis thaliana* accessions from Cape Verde Islands

**DOI:** 10.1126/sciadv.adq8210

**Published:** 2025-07-11

**Authors:** Félix J. Martínez Rivas, Dorothee Wozny, Zeyun Xue, Elodie Gilbault, Thomas Sapir, Melissa Rouille, Antony Ricou, Joaquín Medina, Laurent D. Noël, Emmanuelle Lauber, Aline Voxeur, Marianne Mazier, Olivier Loudet, Gilles Clément, Jose M. Jiménez-Gómez

**Affiliations:** ^1^ Centro de Biotecnología y Genómica de Plantas, Universidad Politécnica de Madrid (UPM)–Instituto Nacional de Investigación y Tecnología Agraria y Alimentaria (INIA/CSIC), Campus de Montegancedo UPM, 28223 Pozuelo de Alarcón, Spain.; ^2^ Departamento de Bioquímica y Biología Molecular, Universidad de Córdoba, Edificio Severo Ochoa, Campus de Rabanales, E-14014 Córdoba, Spain.; ^3^ Université Paris-Saclay, INRAE, AgroParisTech, Institut Jean-Pierre Bourgin (IJPB), 78000 Versailles, France.; ^4^ LIPME, Université de Toulouse, INRAE/CNRS, UMR 0441/2598, F-31326 Castanet-Tolosan, France.; ^5^ GAFL-Génétique et Amélioration des Fruits et Légumes, UR1052, Allée des chênes CS60094, 84143 Montfavet Cedex, France.

## Abstract

Soil salinization poses a notable threat to agriculture. The Cape Verde Islands are located 600 km off the coast of Africa and are characterized by arid environments and high-salinity soils. Here, we find that *Arabidopsis thaliana* plants native to these islands accumulate glucuronyl-mannose that protects them from salt stress. We map the ability to produce this metabolite to an α glycosidase family 38 gene that we named *GH38cv*. Plants carrying mutant alleles of GH38cv do not present defects on growth, fitness, or biotic resistance under standard laboratory conditions but show better germination rates, longer roots, better hydric status, and higher fitness than nonmutated plants when exposed to salt stress. Notably, deleterious mutations in GH38cv have evolved independently on two separate islands within the Cape Verde archipelago, illustrating an example of parallel evolution for salt tolerance in this environment. Our findings reveal a knowledge-driven method to develop salt-resilient crops, which could be attractive to both conventional and organic agriculture.

## Teaser

Plants from the Cape Verde Islands evolved parallel mutations to protect from high salinity.

## INTRODUCTION

Plants colonizing harsh ecological niches can evolve adaptations to local challenges. An especially intriguing example of adaptation is when two lineages independently evolve the same characteristic in response to similar evolutionary pressures, a phenomenon known as parallel evolution (*1*–*3*). Parallel evolution is considered as strong evidence for adaptation by natural selection, because it is unlikely that similar phenotypes would evolve independently multiple times purely by chance. However, other factors, such as differential mutation rates in specific genes, can also produce similar outcomes (*4*). Nevertheless, cases of parallel evolution offer unique opportunities to study adaptation, as they provide natural replicates of the evolutionary process.

Soil salinization is spreading globally and affects 25% of irrigated lands (*5*). Only about 1% of all plant species can tolerate high salt concentrations, complicating cultivation in saline areas such as islands or coastal regions (*6*). The Cape Verde Islands, located approximately 600 km off the west coast of Africa, exhibit high salinity in both soil and inland water sources (*7*, *8*). The Cvi-0 accession of the model plant *Arabidopsis thaliana* was collected from these islands in the 1990s and has been extensively studied due to its unique characteristics, including strong seed dormancy, reduced freezing tolerance, early flowering, high stomatal conductance, and modified root hydraulics (*9*–*12*). In terms of its tolerance to salinity, Cvi-0 is known to germinate better and produce longer lateral roots than the reference accession Col-0 under high salt concentrations (*13*, *14*). Recently, 335 *Arabidopsis* individuals from seven populations on the islands of Santo Antão and Fogo in the Cape Verde Islands were sampled (*15*). These individuals were more closely related to one another than to any accession in the worldwide collection, suggesting that they form isolated populations. Their ancestors were predicted to colonize the island of Santo Antão between 5000 and 7000 years ago, and subsequently the island of Fogo between 3000 and 5000 years ago (*15*). These populations appear to have evolved in isolation and have developed differences in traits such as flowering time, nutrient transport, and water use efficiency (*15*–*18*).

Plant responses to high salinity have been well characterized at both the physiological (*19*) and molecular levels (*20*). High salinity induces the production of reactive oxygen species in cells, leading to oxidative stress (*21*). Typical responses include the accumulation of toxic ions, impaired nutrient uptake, reduced cell turgor, stomatal closure, and altered photosynthesis (*20*). To reduce water loss and enhance cell turgor under saline stress, plants can accumulate osmoprotectants such as proline, soluble sugars, glycine betaine, and polyols (*22*). These metabolites can be precisely detected and quantified with techniques such as liquid and gas chromatography–mass spectrometry (LC-MS and GC-MS, respectively) (*23*, *24*). The most common signatures detected in GC-MS profiles can be identified by comparison with mass spectral databases, which are continuously expanding. However, many detected features remain uncharacterized, presenting great opportunities for discovery of novel compounds.

The likelihood to identify unknown metabolites increases when analyzing divergent plant populations from different regions of the world, or those adapted to different environments. When population-specific metabolites are discovered, their genetic basis can be identified through association mapping. In such studies, metabolomic profiling is performed on plant populations that segregate for the metabolite of interest, and the association of allelic variation with metabolite abundance enables localization of the causal mutation to a chromosomal region (*25*–*31*). If the metabolite under study confers an advantage under specific environmental conditions, identification of the precise mutations, genes, and pathways involved can be useful to synthesize the metabolite for its use in bio-fortification or bio-protection, or to perform gene editing of the locus in plants of societal interest.

Here, we conducted metabolic profiling in a population derived from a cross between the reference accession Col-0 and the Cape Verde Islands accession Cvi-0. We identified a disaccharide not present in existing spectral mass databases and characterized it as glucuronyl-mannose. We then mapped the causal mutation to the α-mannosidase gene *AT3G26720*, which has previously been implicated in salt stress. We confirmed that mutations in *AT3G26720* and the resulting increase in glucuronyl-mannose production enhance *Arabidopsis* tolerance to salt stress. Furthermore, our analysis of the evolutionary history of *AT3G26720* revealed two independent mutant alleles, each specific to a different island in the Cape Verde archipelago, constituting a case of parallel evolution toward the production of the metabolite in this environment.

## RESULTS

### Metabolite variation and QTL mapping in the Col-0 × Cvi-0 RIL population

We explored metabolite variation in a recombinant inbred line (RIL) population derived from the refence *A. thaliana* accession Col-0 (from central Europe) and Cvi-0 (from the Cape Verde Islands) (*32*) (dataset S1). To ensure robustness of the metabolic dataset, four replicates of each line were grown in two consecutive and independent experiments in the Phenoscope, a robotic platform that automatically waters plants and captures images several times a day (*33*). The aerial part of each plant was collected after 23 days on the robot (31 days after sowing), and metabolites were quantified using GC-MS. We identified 118 metabolites that were present in more than 45 RILs (half the lines in the population; dataset S1). Clustering of the metabolic profiles across the population revealed strong correlations among primary metabolites, and between metabolites in the tricarboxylic acid (TCA) trichloroacetic acid cycle (fig. S1).

**Fig. 1.**
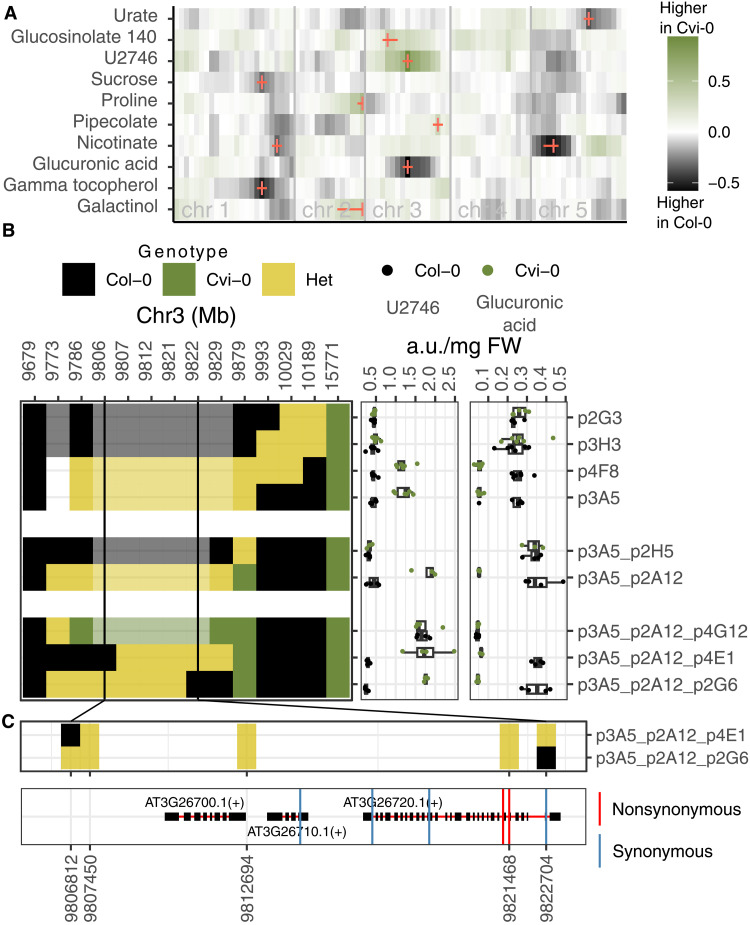
Mapping and fine mapping of metabolite QTLs in Cvi-0 × Col-0. (**A**) Heatmap with QTL effects for the top 10 metabolite QTLs in the population. Markers are placed along the *x* axis, separated by chromosomes by vertical gray bars. The color scale in the heatmap represents the calculated additive effects of the homozygous alleles. The marker with the highest LOD score for each trait is marked with colored vertical bars. Confidence intervals for each QTL are indicated with horizontal red lines. (**B**) Left panel shows the genotypes of recombinant plants selected from HIF474 (*y* axis) for markers at the indicated positions in chromosome 3 (*x* axis). Solid and light coloring represents real and imputed genotypes, respectively. Vertical black lines indicate the region delimiting the candidate mutation. Right panel shows the abundance of U2746 and glucuronic acid in the progenies inheriting Col-0 (black) or Cvi-0 alleles from the recombinant plant with genotypes shown in the left. a.u., arbitrary units. (**C**) Top panel shows the genotype and physical position of the markers tested in the two recombinants that delimit the causal region. Bottom panel shows the genes in the causal region and the position of all coding mutations between Col-0 and Cvi-0.

We used mixed-effect models to estimate metabolite abundance in RIL while correcting for batch effects from GC-MS injection (see Materials and Methods). These corrected values were used for quantitative trait locus (QTL) analysis. We identified 44 QTLs associated with 35 metabolites, an average of 1.25 QTLs per metabolite with a detectable genetic basis (figs. S2 and S3A and dataset S2). QTL hotspots were identified in chromosomes 1 and 3 (fig. S3B), with Cvi-0 alleles at both hotspots increasing the concentration of metabolites such as aconitate, citrate, maleate, phosphate, and myo-inositol-1-P (figs. S2 and S3). Among the top 10 QTLs with the larger effects (Fig. 1A), two had been previously described: one for proline in chromosome 2, and another for a glucosinolate derivate in chromosome 3 (*34*, *35*). The two QTLs with the largest effects were located in chromosome 3 and affected the levels of glucuronic acid and another metabolite with a retention index of 2745.9 and a characteristic ion with a mass/charge ratio (*m*/*z*) of 494. We designated this unknown metabolite as U2746 based on its retention index. In the RILs, the Cvi-0 allele at the U2746 QTL decreased the abundance of glucuronic acid and increased the levels of U2746, suggesting that a single causal mutation may control both metabolites.

### Fine mapping of the U2746 QTL

To fine-map the mutation underlying U2746 accumulation in Cvi-0, we grew three heterogeneous inbred families (HIFs). These are RILs that carry a single heterozygous genomic fragment overlapping the QTL region (*36*). The progeny of two HIFs showed differences in the accumulation of U2746 associated to contrasting alleles of the QTL (fig. S4). We screened 1440 descendants from one of these HIFs (8HV474) during three rounds of fine mapping, narrowing the QTL region down to three genes (Fig. 1B). Throughout these experiments, metabolic profiling consistently showed opposite concentrations of U2746 and glucuronic acid in lines carrying homozygous Col-0 and Cvi-0 alleles at the QTL (Fig. 1B). Among the three candidate genes, one encodes a glycoside hydrolase family 38 protein (*AT3G26720*, hereafter named *GH38cv*). This protein has been described to function as an α-mannosidase (*37*), and its mutation increases salt tolerance in both *Arabidopsis* and wheat (*38*, *39*). We selected homozygous descendants from HIF line p3A5_p2A12_p4E1 in Fig. 1B for further validation and characterization of the QTL, hereafter referred to as HIF-Col and HIF-Cvi.

### GH38cv underlies the U2746 QTL

**Fig. 2.**
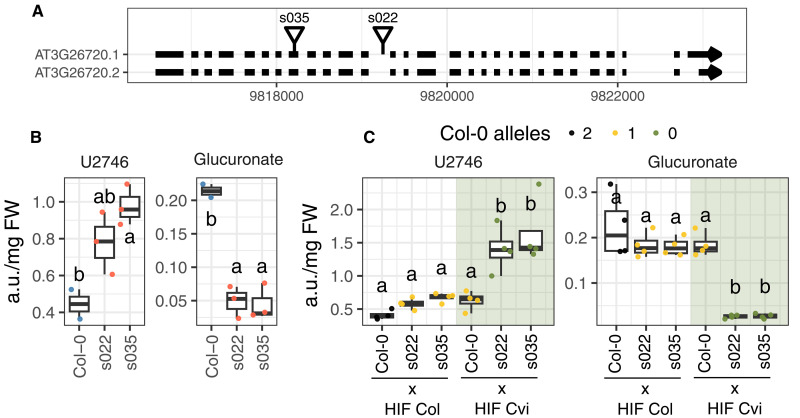
Confirmation of the effect of AT3G26720 on metabolite accumulation. (**A**) Genomic positions of T-DNA insertions in AT3G26720 based on coordinates from Salk Signal T-DNA Express in the TAIR10 genome reference and annotation. Black boxes are exons; arrows point to the direction of transcription. s035 stands for line SALK_035007C and s022 for SALK_022207C. (**B**) Normalized abundance in arbitrary units of U2746 (left) and glucuronic acid (right) for Col-0 and two T-DNA insertion mutants. (**C**) Normalized abundance in arbitrary units of U2746 (left) and glucuronic acid (right) for hybrid plants resulting from crosses of the specified lines in the *x* axis. Different letters represent significant differences between lines [one-way analysis of variance (ANOVA), Tukey HSD test, *P* < 0.05]. Shaded area in each plot indicates lines that carry one Cvi-0 allele, and nonshaded areas indicate lines with no Cvi-0 alleles. Dot colors mark the number of Col-0 alleles in each line.

We first confirmed the causal role of GH38cv in U2746 accumulation using two independent T-DNA insertion lines in a Col-0 background. Both lines exhibited increased U2746 levels and decreased glucuronic acid compared to Col-0 (Fig. 2, A and B). One of the mutants had an insertion in an intron and showed lower levels of U2746 than the mutant with an insertion in an exon, which agrees with the latter being more disruptive of the activity of the gene since it changes its amino acid sequence (*40*).

We then used quantitative complementation to confirm the differential effect of the Col-0 and Cvi-0 alleles of GH38cv on the accumulation of U2746 and glucuronic acid. For this, we crossed the T-DNA insertion mutants with the homozygous HIF-Col and HIF-Cvi lines and obtained plants displaying zero, one, or two copies of the Col-0 allele, and zero or one copy of the Cvi-0 allele. Plants carrying more copies of *GH38cv* Col-0 allele showed variation in U2746 and glucuronic acid accumulation, while the number of Cvi-0 alleles had no effect (Fig. 2C), suggesting that the Cvi-0 alleles of GH38cv are functionally null. In summary, these experiments indicate that GH38cv directly controls both U2746 and glucuronic acid levels, and that the Cvi-0 allele harbors one or several mutations compromising its function.

### Characterization of the U2746 metabolite

**Fig. 3.**
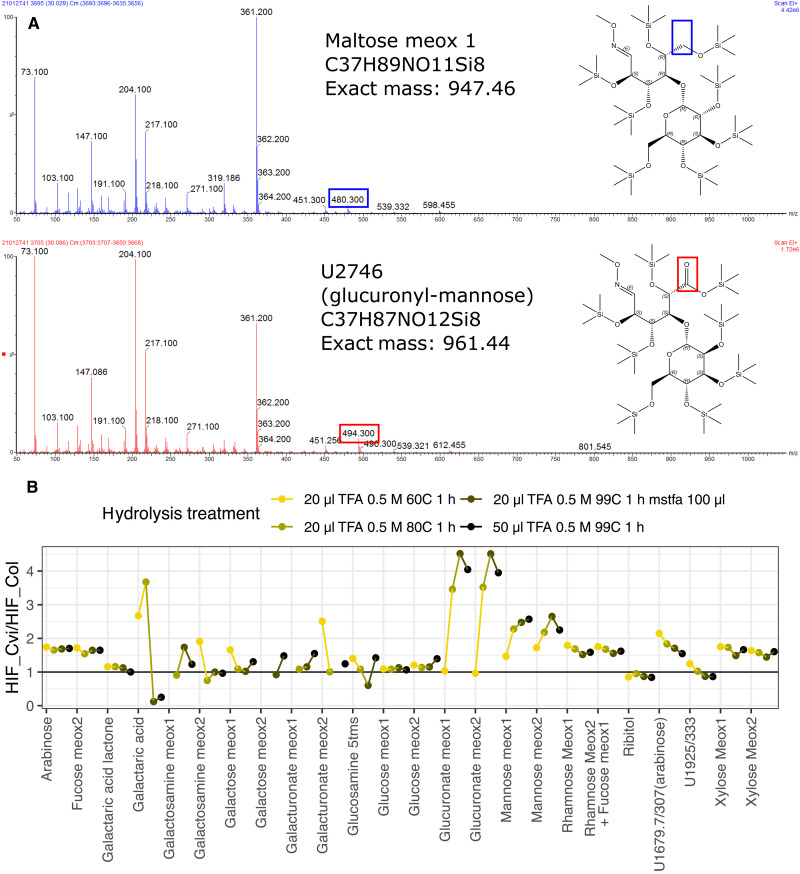
Characterization of U2746. (**A**) EI 70ev spectra for maltose (top) and U2746 (bottom) from *m*/*z* 50 to 1050 from leaf extracts of HIF-Cvi. The main difference in these spectra is marked with a box. This 14-Da difference fits with U2746 having an extra oxygen, marked in the representation of the molecules to the right of the spectra. (**B**) Ratio of sugars released after acid hydrolysis of leaf extracts from HIF-Col and HIF-Cvi. Different color points from yellow to dark indicate stronger hydrolysis conditions. From all sugars detected, only glucuronic acid and mannose appear in higher concentrations in HIF-Cvi than in HIF-Col with stronger TFA treatment, suggesting that U2746 is glucuronyl-mannose.

We then analyzed the chemical nature of U2746 and its relation with glucuronic acid. GC-MS data analysis revealed similar retention index and mass spectra between U2746 and the disaccharide maltose (fig. S5). Their fragmentation spectra differed in the shift of a peak in U2746 from *m*/*z* 480 to *m*/*z* 494, representing a 14-Da difference that could be explained by the presence of uronic acid as one of the saccharides (see molecule representation in Fig. 3A). We confirmed the disaccharide nature of U2746 in leaf extracts form HIF-Col and HIF-Cvi using acid hydrolysis of ether bonds with increasing strengths (Fig. 3B). Methoxyamine derivatives of glucuronic acid and mannose appeared as the hydrolysis treatments become stronger, suggesting that U2746 is composed of these two sugars linked by an ether bond.

We then purified leaf extracts from Col-0 and one of the T-DNA insertion lines with an anion exchange column. U2746 eluted with organic acids, proving that it contains an acidic moiety (fig. S6A). Trifluoroacetic acid hydrolysis of the partially purified sample released neutral and acidic monosaccharides, with significant enrichment of mannose and glucuronic acid in the T-DNA insertion line (hexoses in fig. S6B), supporting again that U2746 is glucuronyl-mannose (fig. S6C). In the metabolomic data obtained during the QTL analysis, the RILs that carried GH38cv Cvi-0 alleles had significantly higher levels of the U2746 metabolite and lower levels of glucuronic acid than the lines carrying GH38cv Cvi-0 alleles (fig. S7, A, B, and D). On the contrary, only slight, nonsignificant, differences in mannose content were found between lines with contrasting alleles of GH38cv (fig. S7, C and E). One possibility for the lack of association between U2746 and mannose is that, once U2746 is cleaved into mannose and glucuronic acid by GH38cv, mannose is metabolized faster than glucuronic acid, for example, toward ascorbic acid synthesis (*41*). Another small but feasible possibility is that mannose is replaced by another hexose that could not be detected in the eluate of the exchange columns. Together, our data suggest that U2746 is an ether-linked glucuronyl-mannose, although determining the exact structure of the metabolite would need further investigation.

### Functional characterization of GH38cv

To assess the role of GH38cv during development, we tracked U2746 and glucuronic acid levels over time. We grew the HIF lines with contrasting allele of GH38cv in a growth chamber under long day conditions until senescence. The concentration of U2746 increased with plant age in both HIF-Cvi and HIF-Col, but it increases up to four times more in HIF-Cvi (fig. S8). The concentration of glucuronic acid showed the opposite pattern, increasing in HIF-Col but not in HIF-Cvi. The concentration of mannose remained virtually constant during plant development until senescence (fig. S8).

**Fig. 4.**
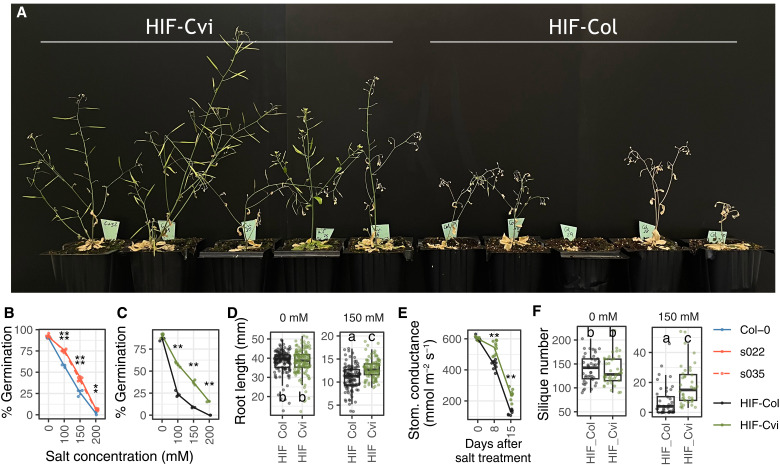
Effect of mutations in AT3G26720 in response to high salinity. (**A**) HIF-Col and HIF-Cvi plants after 7 days of irrigation with salted water. (**B** and **C**) Germination after 7 days in different concentrations of salt in seeds from T-DNA insertion lines (B) or HIF lines with contrasting alleles of AT3G26720. Asterisks indicate significant differences at a given salt concentration between Col-0 and the T-DNA insertion mutants in (B) or Col-0 and Cvi-0 alleles in the HIFs in (C) (Student’s *t* test, **P < 0.01, *P < 0.05). Data are from two independent experiments. (**D**) Root length 5 days after transfer to control (0 mM) or salt medium (150 mM) in the HIF lines with contrasting GH38cv alleles. Data are from four independent experiments. Different letters indicate significant differences between lines and conditions (two-way ANOVA, Tukey HSD test, *P* < 0.05). (**E**) Stomatal conductance in plants treated with salted water. Treatment starts at day 0. Asterisks indicate significant differences at a given day (Student’s *t* test, **P < 0.01). (**F**) Silique number in plants irrigated with nonsalted water or salted water (*P* control = 0.512, *P* salt = 7.15 × 10^−5^).

The *GH38cv* gene was first identified in wheat through its binding to an E3 ligate implicated in salt tolerance (*39*). The *Arabidopsis* T-DNA insertion lines for *GH38cv* showed increased germination rate, root length, and fresh weight under high salinity (*38*, *39*). We evaluated salt tolerance in our lines and confirmed these previous results (Fig. 4A). Both the T-DNA insertion lines and HIFs segregating for *GH38cv* Cvi-0 and Col-0 alleles showed increased germination rates (Fig. 4, B and C). The similar phenotypes in both T-DNA and HIF lines support again the lack of function of the GH38cv Cvi-0 allele. In addition, HIF-Cvi had longer roots than HIF-Col under high salt concentrations (Fig. 4D) and observed that adult HIF-Cvi plants had higher stomatal conductance and increased fitness than HIF-Col when exposed to salt stress (Fig. 4, A, E, and F, and fig. S11). Despite all their differences under high salinity, no differences were observed between the lines under control conditions (Fig. 4 and fig. S11), suggesting that GH38cv mutations enhance salt tolerance without developmental penalties under standard laboratory conditions. Such a system could be a putative solution for growing plants under high salinity in agriculture without compromising plant performance.

Following this idea, we studied whether the accumulation of U2746 affected plant growth. Image analysis from the Phenoscope experiments allowed determination of 11 descriptors including projected rosette area, convex hull area, circle radius, compactness, growth rate, and color mean and variance. RILs with Cvi-0 *GH38cv* alleles had slightly larger rosettes and faster growth (fig. S9), although these differences were minor and not visible in HIFs or T-DNA lines. In addition, differences in growth rate disappeared when corrected by rosette size, indicating that they were due to larger size of plants carrying Cvi-0 alleles (fig. S9E). GH38cv mutations in both the HIFs and the T-DNA mutants also had no detectable effect on resistance to virulent bacteria (*Xanthomonas campestris* pv. campestris) or fungi (*Botrytis cinerea*) (fig. S10). Together, our results allow us to conclude that the differential accumulation of U2746 due to mutations in GH38cv confers plants with an advantage under high salt concentrations but does not compromise overall plant germination, growth, or responses to *Xanthomonas* and *Botrytis* under standard laboratory conditions.

### Population genetics of GH38cv

We then studied the origin of the *GH38cv* allele from the Cape Verde Islands. Cvi-0 carries two nonsynonymous mutations in GH38cv relative to the Col-0 reference: a tyrosine to histidine change at position 773 of the protein (Y773H), and a glycine to aspartic acid change at position 814 (G814D) (Fig. 1). Both positions are invariable in orthologous genes from 11 Brasicaceae species, but position G814D is more conserved than Y773H across a diverse set of 98 plant species (fig. S12). In addition, the G814D mutation is located in a predicted protein cavity (fig. S13), and the G to D change implies differences in size and charge, which could introduce structural strain or alter the local electrostatic environment in GH38cv. Moreover, the G814D change has a SIFT score of 0, indicating the maximum deleterious potential, making it a good candidate to alter the function of GH38cv.

**Fig. 5.**
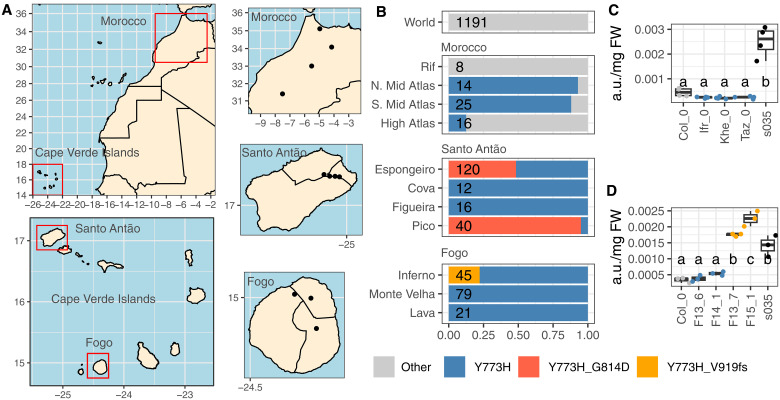
Distribution in Cape Verde Islands and Morocco of variants of interest in GH38cv. (**A**) Maps with geographic regions of interest. Dots indicate the places the populations were collected. (**B**) Frequency of variants Y773H, G814D, and V919fs found for GH38cv in the populations shown in Morocco, Santo Antão, and Fogo. Alleles not presenting any of these variants are called “Other.” The number of individuals in each population is shown at the bottom of each bar. (**C** and **D**) U2746 abundance in arbitrary units in three accessions from Morocco (Ifr-0, Khe-0, and Taz-0) (C) or four accessions from the island of Fogo (F13_6, F14_1, F13_7, and F15_1, with contrasting alleles for the V919fs mutation) (D). Col-0 and the T-DNA insertion line s035 were used as controls. Different letters indicate significant differences between lines (one-way ANOVA, Tukey HSD test, *P* < 0.05). The colors in (B) to (D) indicate whether the accessions carry the Y773H mutation alone, the Y773H and G814D mutations, the Y773H and V919fs mutations, or none.

We screened short-read sequences from 1607 worldwide *Arabidopsis* accessions to explore the distribution of the G814D and T773H mutations (*42*). These accessions include 335 individuals from the Cape Verde Islands (*15*), 64 from Morocco (*43*), and 175 from the Iberian peninsula (*42*, *44*). Both the Y773H and G814D mutations are only present in African accessions (Fig. 5 and dataset S3). The Y773H substitution is present in 50% of the accessions from Morocco but in 100% of the accessions from the Cape Verde Islands, suggesting that it predates the colonization of the Cape Verde Islands (Fig. 5B). The G814D mutation was only found in Santo Antão, suggesting that it originated in this island (Fig. 5B). The mutation is present at high or medium frequency in two of the four populations sampled in the island. In one of the populations (Espongeiro), we found one plant carrying the mutation in heterozygosity, probably due to natural hybridization events that occur at low frequency in *Arabidopsis* (*45*). Moroccan accessions carrying Y773H did not accumulate U2746, while plants carrying both Y773H and G814D did (Fig. 5C), suggesting that the G814D mutation in GH38cv is the most likely cause of accumulation of U2746 in Cvi-0.

We investigated additional mutations in *GH38cv* by mining short-read sequences from other *Arabidopsis* accessions worldwide (*15*, *42*, *43*). We reconstructed in silico 92 different alleles of *GH38cv* (fig. S14) and identified one predicted nonfunctional allele that carries a 1–base pair deletion producing a shift in its open reading frame (V919fs mutation, allele a21 in fig. S14). This allele also originates from the Cape Verde Islands, albeit from the island of Fogo instead of the island of Santo Antão (dataset S3). The frameshift allele was present in 10 of the 45 individuals from the Inferno population of Fogo (Fig. 5B) and was not present in any of the 100 individuals from the other two populations in the island. Individuals from Fogo carrying the V919fs mutation showed significantly higher concentrations of U2746 than other plants from the same island without the mutation (Fig. 5D).

We then studied the distribution of the G814D or V919fs mutations in *GH38cv* among accessions from Santo Antão, Fogo, and Africa. Phylogenetic analyses revealed three distinct populations (fig. S16A). As published before (*15*, *18*), individuals from Santo Antão and Fogo were phylogenetically distinct to those from mainland Africa, and highly similar to individuals from their own island. While the G814D variant is found in multiple clades inside Santo Antao, the V919fs mutation is present in a single clade, suggesting that this frameshift mutation arose later than the G814D mutation (fig. S16B). In addition, the segregation of both mutations among phylogenetically close individuals that are distant to accessions from mainland Africa suggests that both the G814D and V919fs mutations arose independently on the islands of Santo Antão and Fogo, respectively.

We confirmed the effect in salt tolerance of the G814D and V919fs mutations in the genetic backgrounds where these mutations occurred. For this, we measured root length under high salt concentrations in accessions from Morocco, Santo Antão, and Fogo carrying functional and nonfunctional alleles of GH38cv (fig. S15). Accessions carrying the G814D or V919fs mutations presented similar root lengths under control conditions than accessions carrying only the Y773H mutation, but showed longer roots under salt treatment (fig. S15). Together, our data support a model of parallel evolution of mutations in GH38cv, leading to the accumulation of U2746 to increase salt tolerance in *Arabidopsis* plants from the Cape Verde Islands.

## DISCUSSION

High salinity in soil and irrigation water negatively affects plant growth and crop productivity. We investigated natural strategies adopted by plants to colonize the arid soils on the Cape Verde Islands and found that accumulation of U2746 enhances salt tolerance. This metabolite is typically present at low concentration in most *Arabidopsis* accessions, but significantly accumulates in GH38cv loss-of-function mutants.

The GH38cv/U2746 combination may influence salt tolerance in *Arabidopsis* through multiple mechanisms. Although GH38cv remains largely uncharacterized in *Arabidopsis*, its homolog in *Brachypodium distachyon* has been shown to accumulate in the endoplasmic reticulum and in the nucleus (*34*). This contrasts with proteomic studies summarized in the SUBA5 database, which report its secretion to the cell wall (*46*). If this is accurate, the α-mannosidase activity of GH38cv could target mannose-rich carbohydrates, such as hemicelluloses, for cell wall remodeling or degradation. Glucuronic acid is a key component of xylan, the main structural hemicelluloses in plant cell walls (*47*), and xylan content has been shown to decrease in the wall in response to salt stress (*48*). It is possible that accumulation of glucuronyl mannose in Cape Verde Island accessions reduces the availability of glucuronic acid for proper cell wall modeling, thereby affecting salt tolerance. Functional studies characterizing GH38cv’s activity, substrate specificity, and subcellular localization will help elucidate the mechanisms underlying the altered carbohydrate profile observed in gh38cv mutants.

An alternative mechanism of salinity tolerance may involve glucuronyl-mannose acting as an osmoprotectant. Other disaccharides, such as trehalose, are known to play key roles in salinity stress (*49*). Trehalose has been detected at high concentrations in halophyte plants, where it appears to function as an osmolyte (*50*), increasing water uptake potential and protecting cellular membranes from oxidative damage (*22*). In contrast, in nonhalophyte plants, trehalose is found at very low levels and is thought to act as a signaling molecule, although direct evidence is lacking (*51*, *52*). Nonetheless, both exogenous application and endogenous production of trehalose have been effective in enhancing salinity tolerance in several economically important species, including wheat, rice, maize, or tomato (*52*–*55*). In our experiments the peak areas of U2746 and maltose were within the same order of magnitude in arbitrary units (fig. S5). In typical *Arabidopsis* leave samples, maltose is quantified at approximately 3.5 ng/mg fresh weight (1.02 × 10^−11^ mol/mg). We estimate the abundance of U2746 to be in a similar range, given that both molecules are disaccharides with likely comparable response coefficients. Thus, the concentration of U2746 appears too high for a signaling molecule and too low for a classical osmolyte. Further research is necessary to determine whether glucuronyl-mannose functions as an osmoprotectant, a cell wall modifier, a signaling molecule, or potentially all of these.

Here, we discover that the accumulation of glucuronyl-mannose has evolved independently twice in *Arabidopsis* populations from the Cape Verde Islands. The island’s location far from the coast, volcanic soils, harsh climatic conditions, and relatively early colonization by *Arabidopsis* offer a valuable system to study adaptation to extreme environments. Previous works with Cape Verde populations have revealed cases of parallel evolution in traits such as early flowering and nutrient transport (*15*, *16*). In both cases, independent mutations in flowering time genes like *FRI* or *FLC*, and nutrient transport genes like *IRT1* and *NRAMP1*, contributed to the emergence of new adaptive phenotypes in the islands. We report a similar case of parallel evolution, although in a single gene (*GH38cv*). Genomic regions harboring high frequencies of adaptive mutations have been documented in sticklebacks, plants, and other organisms (*56*–*58*), and allelic heterogeneity has also been reported among local *Arabidopsis* populations (*59*, *60*). At the evolutionary level, such cases are often interpreted as evidence of positive selection driving local adaptation (*57*). In our study, although the repeated mutation of GH38cv leading to U2746 accumulation and enhanced salinity tolerance in Cape Verde populations is consistent with this pattern, we currently lack direct evidence of positive selection, and further experiments are required to test this hypothesis and determine whether the observed genetic changes confer a selective advantage in nature.

Our findings might have practical implications for agriculture. Disaccharides like trehalose can be applied exogenously to improve crop tolerance to soil salinity (*52*–*55*). Additionally, genome editing of GH38cv homologs could enhance salinity tolerance in globally important crop, as GH38cv homologs are present in numerous plant families (fig. S12). In wheat, GH38cv interacts with the U-box E3 ubiquitin ligase TaPUB1, and its expression is induced by salt treatment (*39*). Overexpression of GH38cv in *B. distachyon* led to decreased salinity tolerance, supporting its involvement in stress responses (*38*, *39*). In summary, both the synthesis and application of glucuronyl-mannose, as well as targeted gene editing of *GH38cv*, offer promising, nature-inspired strategies to enhance salt tolerance in crops and ornamental plants through biotechnological approaches.

## MATERIALS AND METHODS

### Plant growth for QTL analysis and confirmation

A subset of 90 lines from a Cvi-0 × Col-0 RIL population (*32*) was used for metabolite profiling and mapping of metabolomic QTLs. These 90 lines were selected among the core collection (164 lines) to maximize the frequency of Col-0 and Cvi-0 at each marker (table S1). Genotypes were obtained from the Versailles Arabidopsis Stock Center (*61*). All lines were grown in two consecutive experiments in the Phenoscope (https://phenoscope.versailles.inrae.fr/), a robotic platform that continually rotates individual pots across a controlled environment chamber, watering each pot according to their weight (*33*). Two replicates of each RIL were grown in each experiment. For this, seeds were stratified in a 0.1% agar solution for 3 days at 4°C in the dark and sown on 4-cm plugs of peatmoss substrate mix (blond peat, perlite, and sand) wrapped in a nonwoven film. Plugs were maintained at saturation (100% of the maximum soil water content) in plastic trays until germination, and immediately after seedlings were thinned on each plug to retain only one individual, with as much synchronous germination as possible across plugs. These plugs were then installed on the Phenoscope robot 8 days after sowing. From sowing to the end of the experiment, plugs were watered twice a day to maintain the soil at 60% of the maximum water content using nutrient solution containing 5 mM NO_3_^−^, providing a nonlimiting water and nutrient condition.

The photoperiod in the growth room was set to short days (8-hour light/16-hour dark) to avoid interaction with bolting and to optimize the study of vegetative shoot growth during the exponential growth phase. Light was provided by 6500K white light-emitting diode (LED) sources to an intensity of 230 μmol m^−2^ s^−1^. The air temperature was set to 21°C during the day and 17°C at night, with a constant relative humidity of 65%. The complete aerial part of each plant was harvested after 23 days (i.e., 31 days after sowing), flash frozen in liquid nitrogen, and grinded for metabolomics. A total of 360 samples were collected for metabolomics.

For QTL confirmation, descendants from 28 heterogeneous inbred lines (HIFs) were grown in the Phenoscope platform. From each HIF, we grew three replicates from two independent lines carrying Col-0 alleles at the segregating region and two lines carrying Cvi-0 alleles at the segregating region. Additional replicates were grown for three HIFs for which we only had one Col-0 or Cvi-0 line. The conditions in the Phenoscope were identical to the experiment for the QTL analysis. After 23 days on the robot, the complete aerial part of each plant was harvested, flash frozen in liquid nitrogen, and grinded for metabolomics.

Moroccan accessions Ifr-0 and Khe-0 are originally from the population of South Middle Atlas and Taz-0 from North Middle Atlas and were provided by C. Alonso-Blanco. Accessions F13_6, F13_7, F14_1, and F15_1 originally from the Inferno population in the Island of Fogo were provided by A. Hancock.

### Metabolite profiling by GC-MS

Plant material was collected in 2-ml Safelock Eppendorf tubes, weighted, flash frozen in liquid nitrogen, and ground using the Mixer Mill MM 400 (Retsch). Fifty milligrams of powder was transferred into 2-ml Safelock Eppendorf tubes and resuspended in 1 ml of frozen (−20°C) water:acetonitrile:isopropanol (2:3:3) containing Ribitol at 4 μg/ml and extracted for 10 min at 4°C with shaking at 1400 rpm in an Eppendorf Thermomixer. Insoluble material was removed by centrifugation at 20,000*g* for 5 min. Twenty microliters was collected and dried overnight at 35°C in a SpeedVac and stored at −80°C or immediately injected. Three blank tubes underwent the same steps as the samples.

Samples were taken out of −80°C, warmed 15 min before opening, and SpeedVac dried again for 1.5 hours at 35°C before adding 10 μl of methoxyamine (20 mg/ml) in pyridine to the samples. The reaction was performed for 90 min at 28°C under continuous shaking in an Eppendorf thermomixer. Fifty microliters of *N*-methyl-*N*-trimethylsilyl-trifluoroacetamide (MSTFA) (Aldrich 394866-10x1ml) was then added, and the reaction was continued for 30 min at 37°C. After cooling, 45 μl was transferred to an Agilent vial for injection.

Four hours after derivatization, 1 μl of sample was injected in splitless mode on an Agilent 7890A gas chromatograph coupled to an Agilent 5977B mass spectrometer. The column was an Rxi-5SilMS from Restek (30 m with 10-m integraguard column). The liner (Restek #20994) was changed after each series of 24 samples. Oven temperature ramp was 70°C for 7 min and then 10°C/min to 330°C for 5 min (run length, 38 min). Helium constant flow was 0.7 ml/min. Temperatures were the following: injector: 250°C, transfer line: 290°C, source: 250°C, and quadripole: 150°C. Five scans per second were acquired spanning a 50- to 600-Da range. The instrument was tuned with perfluorotributylamine with 69 *m*/*z* and 219 *m*/*z* of equal intensities. Samples were randomized. Four different quality controls were injected at the beginning and end of the analysis for monitoring of the derivatization stability. An alkane mix (C10, C12, C15, C19, C22, C28, C32, C36) was injected in the middle of the queue for external retention index calibration. Injection volume was 1 μl. The instrument was an Agilent 7890B gas chromatograph coupled to an Agilent 5977B mass spectrometer. The column was an Rxi-5SilMS from Restek (30 m with 10-m integraguard column). The liner (Restek #20994) was changed before the analysis. Oven temperature ramp was 70°C for 7 min and then 10°C/min to 330°C for 5 min (run length, 38 min). Helium constant flow was 0.7 ml/min. Temperatures were the following: injector: 250°C, transfer line: 290°C, source: 250°C, and quadripole: 150°C. Five scans per second were acquired spanning a 50- to 600-Da range. Instrument was tuned with PFTBA with 69 *m*/*z* and 219 *m*/*z* of equal intensities. We performed 43 injections for the samples in the QTL analysis and 9 injections for the samples in the QTL confirmation.

Raw Agilent data files were converted in NetCDF format and analyzed with AMDIS (*62*). A home retention indices/mass spectra library built from the NIST, Golm (http://gmd.mpimp-golm.mpg.de/), and Fiehn databases (http://fiehnlab.ucdavis.edu/19-projects/153-metabolite-library) and standard compounds were used for metabolites identification. Peak areas were also determined with the Targetlynx software (Waters) after conversion of the NetCDF file in masslynx format. AMDIS, Target Lynx in splitless, and split 30 modes were compiled in one single Excel file for comparison. After blank mean subtraction, peak areas were normalized to Ribitol and Fresh Weight.

A response coefficient was determined for 4 ng each of a set of 103 metabolites to the same amount of ribitol. This factor was used to give an estimation of the absolute concentration of the metabolite in what we may call a “one point calibration.” The values obtained for amino acids were checked by a quantification using ion exchange chromatography coupled to post-column derivatization by ninhydrin on an Amino-Tac JLC500 (Jeol) after concentrating 10 times the extracts. The values obtained by both methods were found to be very similar.

The U2746 metabolite was characterized in leaves from HIFs using first partial purification on Anion exchange chromatography and second acidic hydrolysis to determine its composition. Anion exchange chromatography was performed on Sep-Pak Plus Light QMA cartridge (Waters). Cartridges were rinsed with methanol, then 5% ammonium hydroxide, then samples in 5% ammonium hydroxide, then washed with 5% ammonium hydroxide, then washed with methanol, and finally eluted with 2% formic acid in water. Fractions were thoroughly dried in a SpeedVac SPD 111V vacuum centrifuge at 45°C until pressure reached less than 100 μm.

Acidic hydrolysis was performed by adding 30 μl of 0.5 M TFA in water to the dried samples for 1 hour at 99°C under constant shaking at 1400 rpm in an Eppendorf Thermomixer. Samples were thoroughly dried in a SpeedVac SPD 111V vacuum centrifuge at 45°C until pressure reached less than 100 μm. Samples were then processed as described above.

### Metabolite QTL analysis

All metabolomic values are calculated in arbitrary units as described above. For QTL analysis, metabolite quantification values were normalized to the fresh weight of the samples and log_2_ transformed. Metabolomic analysis in the RIL population resulted in abundance values for 112 metabolites. Six metabolites that were absent in more than half of the samples were removed from the analysis. Values from different derivates of the same metabolite were added, and the analysis was performed only for the total amount. We surveyed for metabolite artifacts with the average metabolite concentration of the four replicates for each RIL. Missing values were replaced by the average for the RIL population. Correlation between metabolites was calculated with the heatmap2 function in the gplots package in R (*63*).

We used the lme4 package in R to extract the concentration of each metabolite in each RIL with a mixed effect linear model with injection as a mixed effect factor and genotype as a fixed factor (*64*). We calculated the effect and LOD (logarithm of the odds) score of each marker on each metabolite using the extended Haley-Knott method in the rqtl package (*65*). Thresholds of significance for each metabolite were obtained with a permutation test with 1000 iterations using the extended Haley-Knott method and α of 0.01. We assigned QTL positions as the marker that passed the significance threshold and presented the highest LOD score in the chromosome. We calculated confidence intervals for each QTL using the 1.5 LOD support method implemented in the lodint function from the rqtl package with parameters expandtomarkers = TRUE. QTL effects were calculated using the effectscan function in the rqtl package, which estimates the effect as half the difference between the phenotypic averages for the two homozygotes.

### Fine mapping

We performed three rounds of fine mapping on the progeny of heterogeneous inbred family 8HV474 in the conditions mentioned above, with help from Institut Jean-Pierre Bourgin’s (IJPB) Plant Observatory technological platforms. We screened 400, 400, and 800 plants in each round with polymerase chain reaction (PCR)–based indel, CAPS, or Sanger sequencing markers located in the candidate region (dataset S4).

Plants for fine mapping were grown in 12 cm by 12 cm pots in a temperature-controlled glass house supplemented with ~100 μmol m^−2^ s^−1^ artificial light to ensure 16-hour photoperiods. Leaf tissue was collected in strips of eight 1.2-ml tubes filled with one metal bead, flash frozen in liquid nitrogen, and disrupted in a ball mill for 30 s (Retsch MM400, 30 Hz). DNA extraction was performed according to the method proposed by (*66*). PCRs were performed in a 20-μl volume containing 20 to 40 ng of genomic DNA, 0.25 U of Taq polymerase, 1× supplemented buffer, 0.5 μM forward and reverse primers, and 15 μM deoxynucleotide triphosphates. Bands were visualized on 3% or 4% agarose gels, depending on fragment size differences, containing 4 × 10^–5^ vol/vol % ethidium bromide. At each round, selected recombinant lines were allowed to self and their progeny was screened for homozygous lines. The progeny of these fixed lines was used for metabolic profiling in Fig. 2. Metabolic profiling was performed as described above, but only normalized values of the interesting metabolites were analyzed.

### Physiological characterization of RILs, HIFs, and mutants

Plants from the RIL population were imaged daily during the two consecutive experiments for metabolite QTL analysis in the Phenoscope robotic platform. Images were processed automatically to extract projected rosette area and red/green/blue intensity. Growth rate was calculated as the slope of a generalized linear model using three consecutive projected rosette area values. Significant differences between RILs were calculated with a Student’s *t* test comparing the RILs separated by their genotype at c3_09748, the closest marker to the U2746 QTL.

T-DNA insertion mutant lines in AT3G26720 were obtained from http://signal.salk.edu/ with reference numbers SALK_035007C (hereby called s035) and SALK_022207C (hereby called s022). Predicted insertion sites for these mutants are presented in Fig. 3.

Metabolomic profiling in the T-DNA insertion mutants and the HIFs described in the previous section was performed in plants grown in 12 cm by 12 cm pots in walk-in growth chambers equipped with fluorescent tubes set at 16-hour/8-hour photoperiods and air conditioning set to 21°C/18°C cycles. Leaf samples were taken 2 weeks after flowering, unless otherwise indicated, and processed as described above.

For in vitro analysis, *Arabidopsis* seeds were sterilized in a 2% Tween 20 solution for 5 min, rinsed with water two times, and stratified for 3 days at 4°C in the dark to promote uniform germination. Seeds were then placed in half-strength Murashige and Skoog medium containing 0, 100, 150, or 200 mM NaCl, 1% (w/v) plant agar, and 1% (w/v) sucrose. Plates were then placed in a controlled environment chamber with 16-hour/8-hour light and 21°C/19°C temperature cycles. Plates were scanned in a V600 Epson scanner daily from day 3 to day 7 after sowing. Germination rate was determined by counting the number of germinated seeds from the total number of seeds.

For root growth analysis, seeds were sterilized, stratified, and sowed in plates containing half-strength Murashige and Skoog media as described above. Plates were placed vertically in a growth chamber in the same conditions mentioned above. After 3 days, similar sized plants were transferred either to a Murashige and Skoog agar plate containing 150 mM NaCl or to another Murashige and Skoog agar plate without salt. After 5 days, plates were scanned in a V600 Epson scanner at 400 points per inch and roots were measured using ImageJ.

For analysis of stomata conductance under salt stress, plants were grown in an Aralab walk-in chamber set to 16-hour/8-hour light and 21°C/19°C temperature cycles. After 30 days, plants were watered with 20 ml of a 150 mM NaCl solution every 3 days for 2 weeks. Stomatal conductance was measured every week in fully expanded mature leaves of a total of six to nine plants using a steady-state leaf porometer (SC-1, Decagon Devices, LabFerrer, Spain).

For determining silique number under salinity stress, plants were grown as for stomatal conductance. For determining silique number under control conditions, plants were grown in two independent Aralab walk-in chamber set to 16-hour/8-hour light and 21°C/19°C temperature cycles. One of the chambers was illuminated with LED lights and the other with fluorescent lights. In all experiments, siliques were counted after senescence.

### Infection assays using *Xanthomonas* and *Botrytis*

The virulent *X. campestris* pv. campestris (Xcc) strain 8004∆xopAC (*67*) was grown on MOKA-rich medium (*68*) at 28°C in the presence of rifampicin (50 μg/ml). Infection assays were performed as described (*69*) on 4-week-old *A. thaliana* plants grown in short days (8-hour light) at 22°C (60% relative humidity, 125 μE m^−2^ s^−1^ fluorescent illumination). Fully expanded leaves were wound-inoculated by piercing three times the central vein with a needle dipped in a bacterial suspension at 10^8^ colony-forming units/ml in 1 mM MgCl_2_. Disease development was scored 7 days after inoculation visually in each leaf using the following disease index scale: 0, no symptom; 1, chlorosis at the inoculation site; 2, extended chlorosis; 3, necrosis; 4, leaf death.

For infection with *B. cinerea*, *Arabidopsis* plants were grown in soil in a growth chamber at 22°C, 70% humidity, under irradiance of 100 μmol m^−2^ s^−1^ with a photoperiod of 8-hour light/16-hour dark during 5 weeks. *B. cinerea* B05.10 strain was grown on potato dextrose agar at 23°C under continuous light. After 10 days, each strain produced a dense carpet of conidia. Spores were next washed from the surface of the plate using potato dextrose broth medium. The concentration of spores was determined using a Malassez cell and adjusted to a final concentration of 3.105 conidia/ml. Twenty-microliter drops of spore suspension were placed on *Arabidopsis* leaves of 5-week-old plants. Lesion areas were measured by ImageJ.

### Sequence variation in AT3G26720 in worldwide set of accessions

Sequence variation in AT3G26720 was studied using short-read sequences for 1607 worldwide *Arabidopsis* accessions publicly available at Sequence Read Archive (SRA) (https://www.ncbi.nlm.nih.gov/sra) with accession numbers PRJEB39079 (334 Cape Verde Islands genomes), PRJEB19780 (73 African genomes), PRJNA273563 (1135 worldwide genomes), and PRJNA646494 (65 Iberian Peninsula genomes). Reads were downloaded and aligned to the TAIR10 Col-0 reference using hisat2 v2.1.0 with default parameters except for the --no-spliced-alignment tag (*70*). We discarded reads with mapping quality lower than 5 using samtools v1.7 (*71*). We removed duplicated reads using Picardtools (http://broadinstitute.github.io/picard) and realigned indels using GATK IndelRealigner (*72*). We called variants in all 1607 alignments simultaneously using GATK UnifiedGenotyper (*72*).

For marker design and identification of coding polymorphisms between Col-0 and Cvi-0 in the region of the QTL, we obtained short-read data from Col-0 and Cvi-0 accessions available at SRA (https://www.ncbi.nlm.nih.gov/sra) with reference numbers SRR492239, SRR013327, and SRR013328. We obtained a list of variants between the two accessions using the same mapping, alignment processing, and variant calling methods as above. Primers for small indels and CAPS between Col-0 and Cvi were designed based on the TAIR10 sequence using Primer3 (*73*).

For allele reconstruction, we annotated the predicted effect in the protein of all variants localized in AT3G26720 using ANNOVAR (*74*). We then manually curated the list for possible errors in large effect mutations or heterozygous sites. Eighty-six percent of the heterozygous sites had a minor allele frequency lower than 25% and were supported by less than five reads, so they were converted to homozygous. The rest of heterozygous calls (0.01% of all calls) were discarded. Cases in which two consecutive single-nucleotide polymorphisms (SNPs) were incorrectly annotated were fixed by hand.

For phylogenetic trees in fig. S16, we selected fourfold degenerate SNPs in chromosome 3 with quality above 50 and depth above 30 in all accessions together. We removed variants missing in more than 5% of the accessions and variants in linkage disequilibrium using PLINK with parameters --indep-pairwise 50 10 0.1 (*75*). The remaining 6430 variants were transformed into a tree using the neighbor-joining method from the adegenet and ape packages in R (*76*, *77*). The trees were represented using the ggtree package in R (*78*).

### Statistical analysis

All statistical analyses have been described above.
