# Supplementary material for: Parallel evolution of salinity tolerance in Arabidopsis thaliana accessions from Cape Verde Islands
Source: Sci Adv. 2025 Jul 11;11(28):eadq8210. doi: 10.1126/sciadv.adq8210 (PMC12248283; doi:10.1126/sciadv.adq8210)
Supplement: Supplementary file 1 — Figs. S1 to S16 Legends for tables S1 to S4 [file sciadv.adq8210_sm.pdf]

Science Advances
AAAS

Supplementary Materials for

Parallel evolution of salinity tolerance in *Arabidopsis thaliana* accessions from Cape Verde Islands

Félix J. Martínez Rivas *et al.*

Corresponding author: Jose M. Jiménez-Gómez, jose.jimenez.gomez@csic.es

Sci. Adv. **11**, eadq8210 (2025)
DOI: 10.1126/sciadv.adq8210

The PDF file includes:

Figs. S1 to S16
Legends for tables S1 to S4

Other Supplementary Material for this manuscript includes the following:

Tables S1 to S4

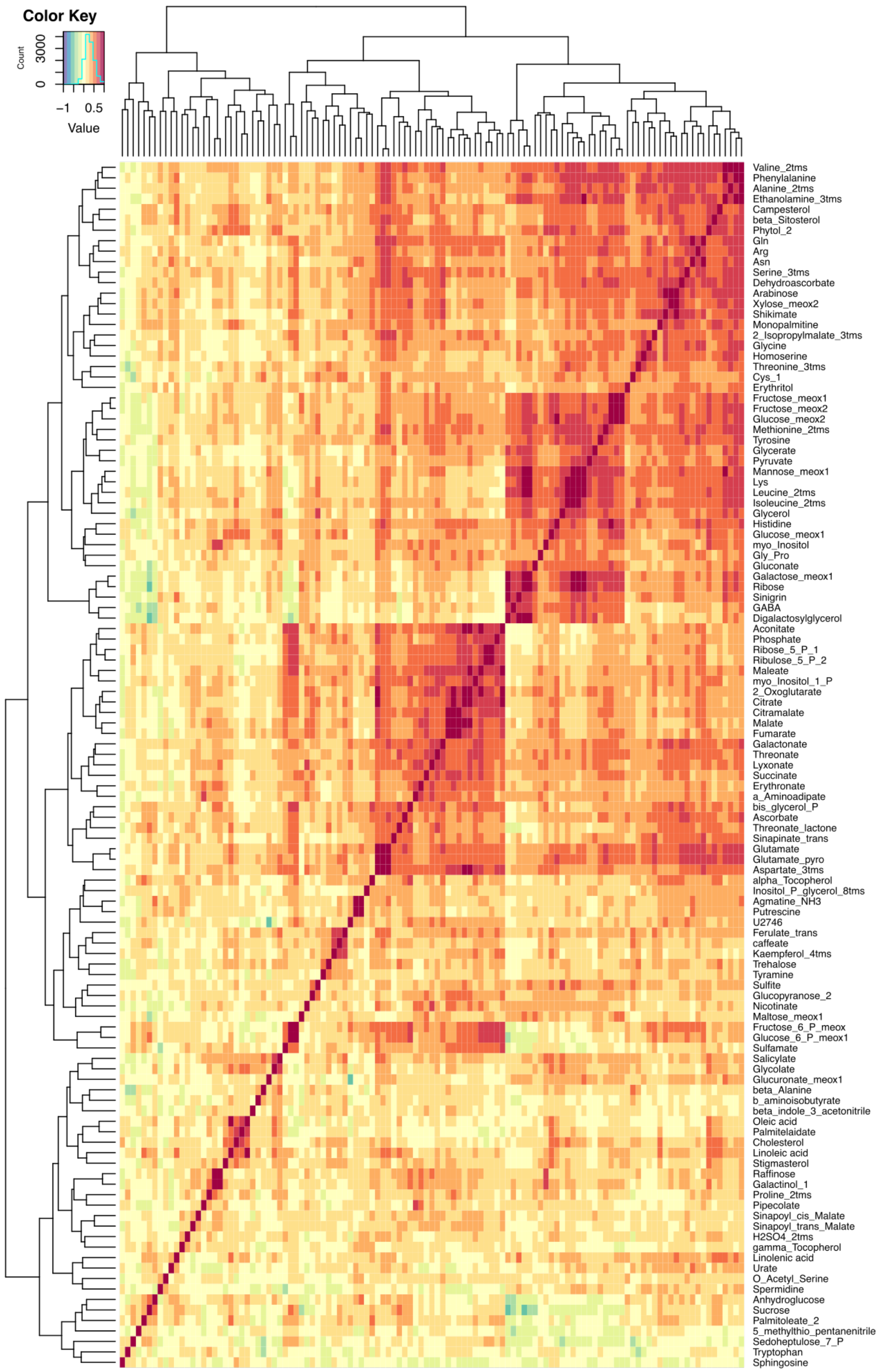

Fig. S1. Metabolite correlation across the RIL population

Correlation heatmap with all metabolites detected in the RIL population. Average metabolite concentration of all replicates for each RIL was used.

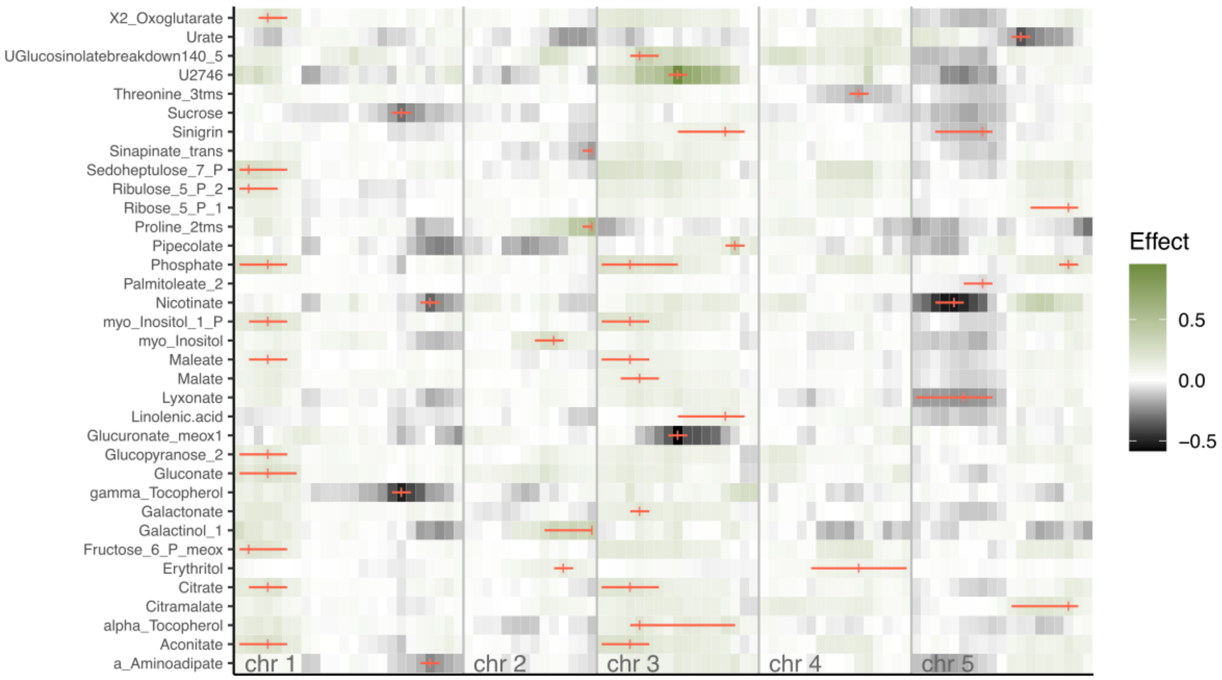

Fig. S2. mQTLs

Heatmap with QTL effects for all metabolites with at least one QTL with LOD>3. Markers are placed along the x axis and chromosomes are separated by vertical gray bars. Rows represent one metabolite. The color scale in the heatmap represent the additive effects of the homozygous alleles, where positive and negative values indicate higher metabolite abundance caused by the Cvi-0 and Col-0 alleles respectively. QTL positions in the heatmap are indicated with small red vertical lines. Horizontal red lines at each QTL represent confidence intervals calculated with the 1.5 LOD support method.

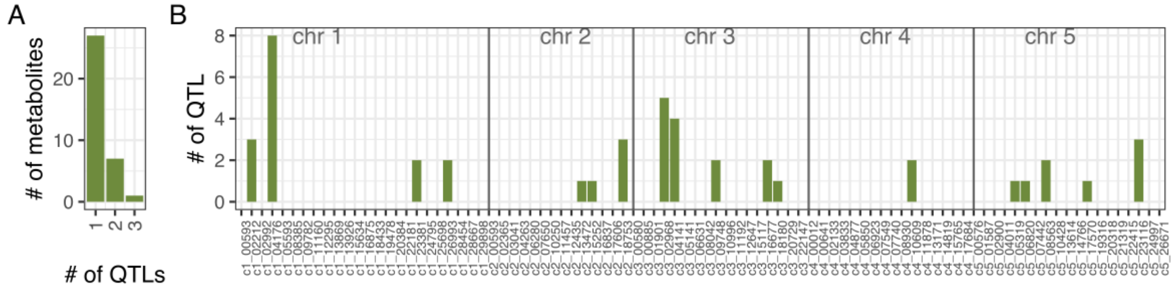

Fig. S3. QTL hotspots
(A) number of QTLs per metabolite with detectable genetic basis (B) Number of QTLs found at each marker position in the RIL population. Chromosomes are separated by gray vertical bars. Marker names are indicated in the x axis.

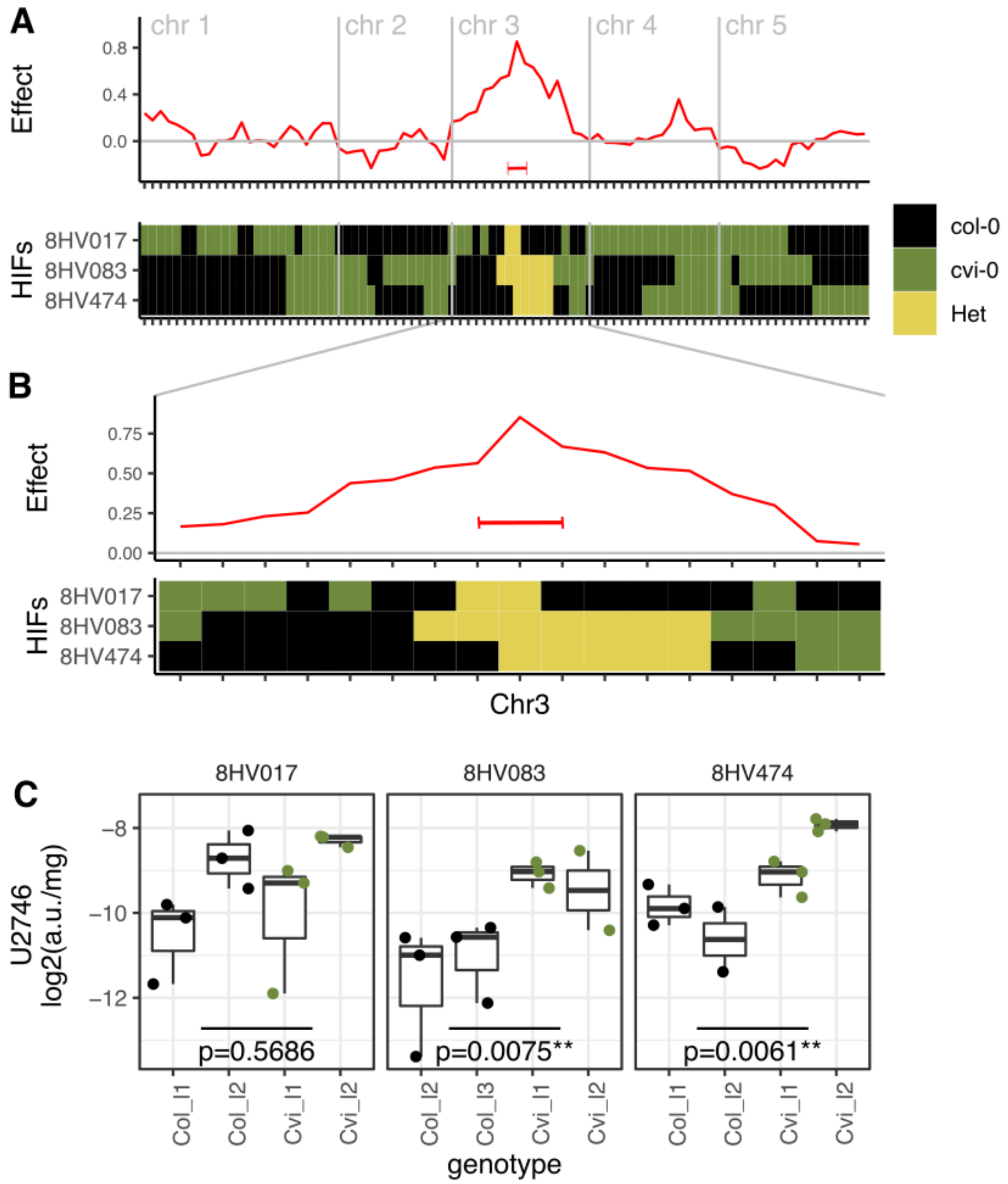

Fig. S4. QTL confirmation using HIFs

(A) Top: Additive effect of homozygous alleles for U2746. Positive values indicate higher U2746 abundance caused by Cvi-0 alleles. Tick marks in the x axis are the markers used for the QTL analysis and HIF genotyping. The horizontal red line shows 1.5 LOD confidence interval for the QTL. Bottom: Genotype for chromosome 3 for three HIFs with a single heterozygous region that overlaps with the U2746 QTL. (B) Zoom on chromosome 3 of the same graphs as above. (C) U2746 abundance in the progeny of each HIF. Y axis represents arbitrary units / mg fresh weight. Plants are homozygous progeny from two independent descendants from each HIF. Numbers in each graph are p values form a one-way ANOVA with genotype as factor. Asterisks mark significant differences between genotypes ($p < 0.01$).

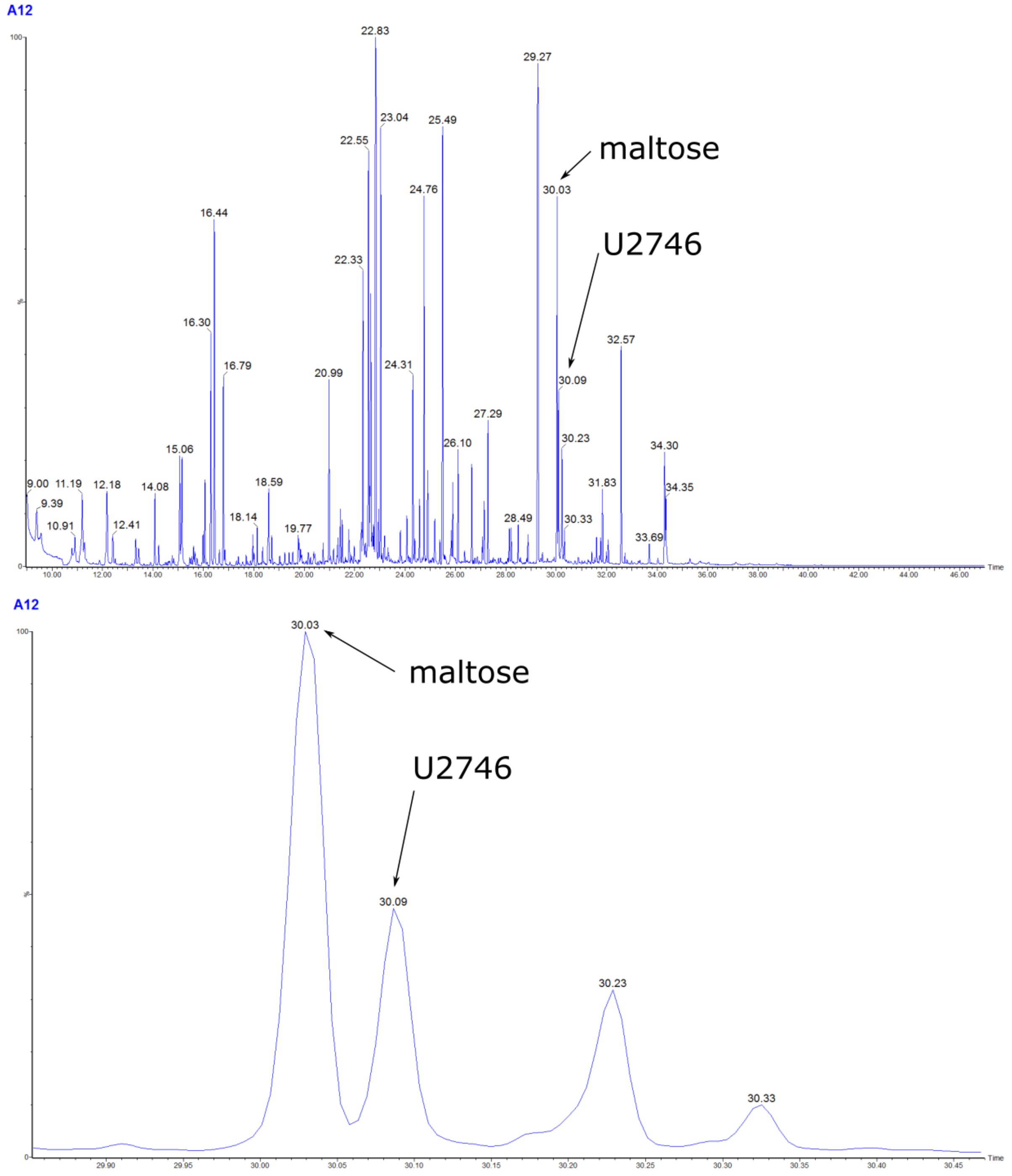

Fig. S5. Retention index U2746

(A) EI 70ev spectra for U2746 and maltose from m/z 50 to 1050 from one plant carrying Cvi-0 alleles at the U2746 QTL in chromosome 3. (B) Zoom-in from (A).

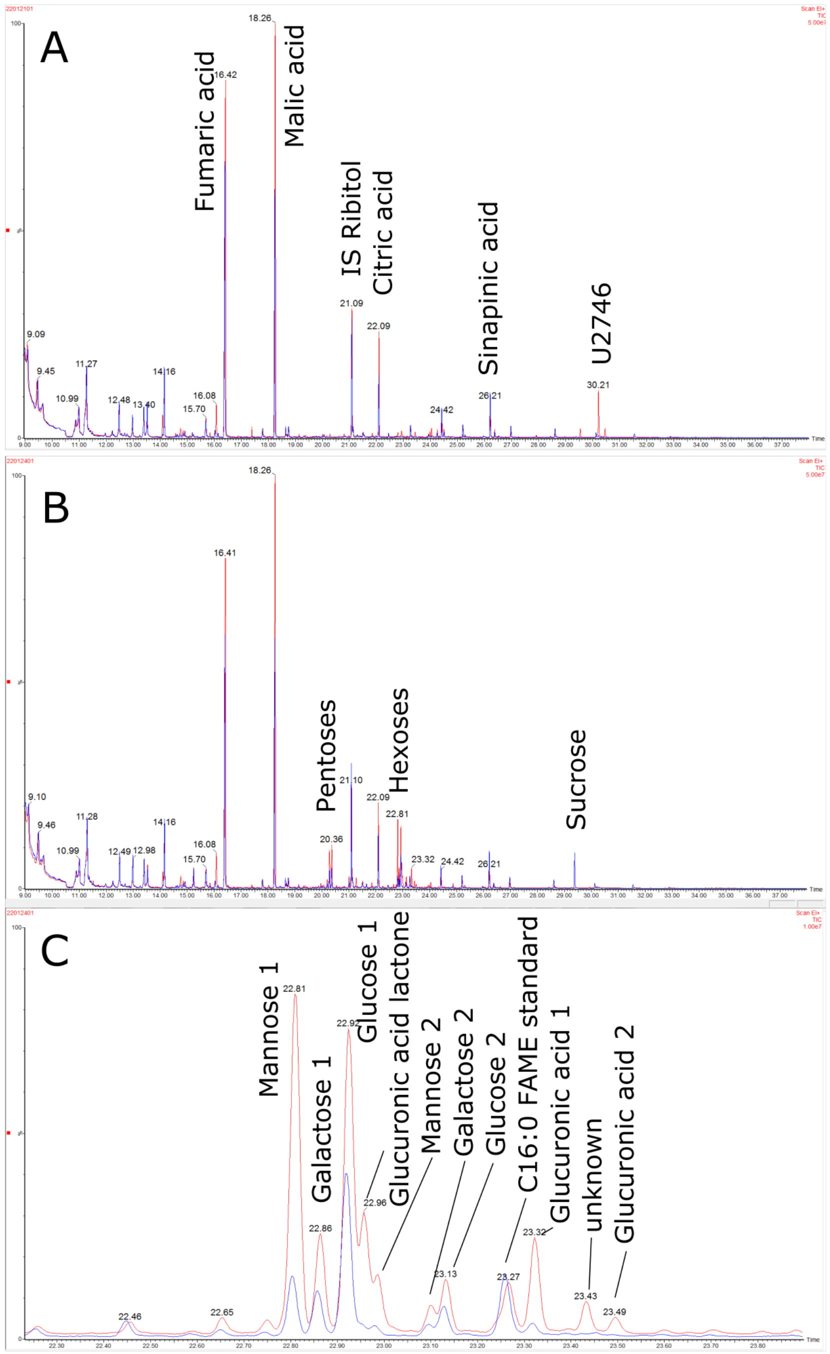

Fig. S6. Fragmentation for U2746

(A) The graph shows the GC-MS EI 70ev spectra from the purification product of the acidic compounds contained in the metabolome extracts of Salk-035 (red, 150mg/ml) and the control Col-0 (blue, 100mg/ml) on a Cation exchange (CEX) Sep-Pak® Plus Light QMA cartridge. (B) 0.5M TFA hydrolysates of the eluates run in (A). (C) Zoom of (B) in the 22.4 to 23.9 min region. Known sugar peaks have been marked with the abbreviation of the sugar name followed by 1 or 2 referring to the first and second methoximated isomers. Man, mannose; Gal, galactose; Glc, glucose; GlcA, glucuronic acid.

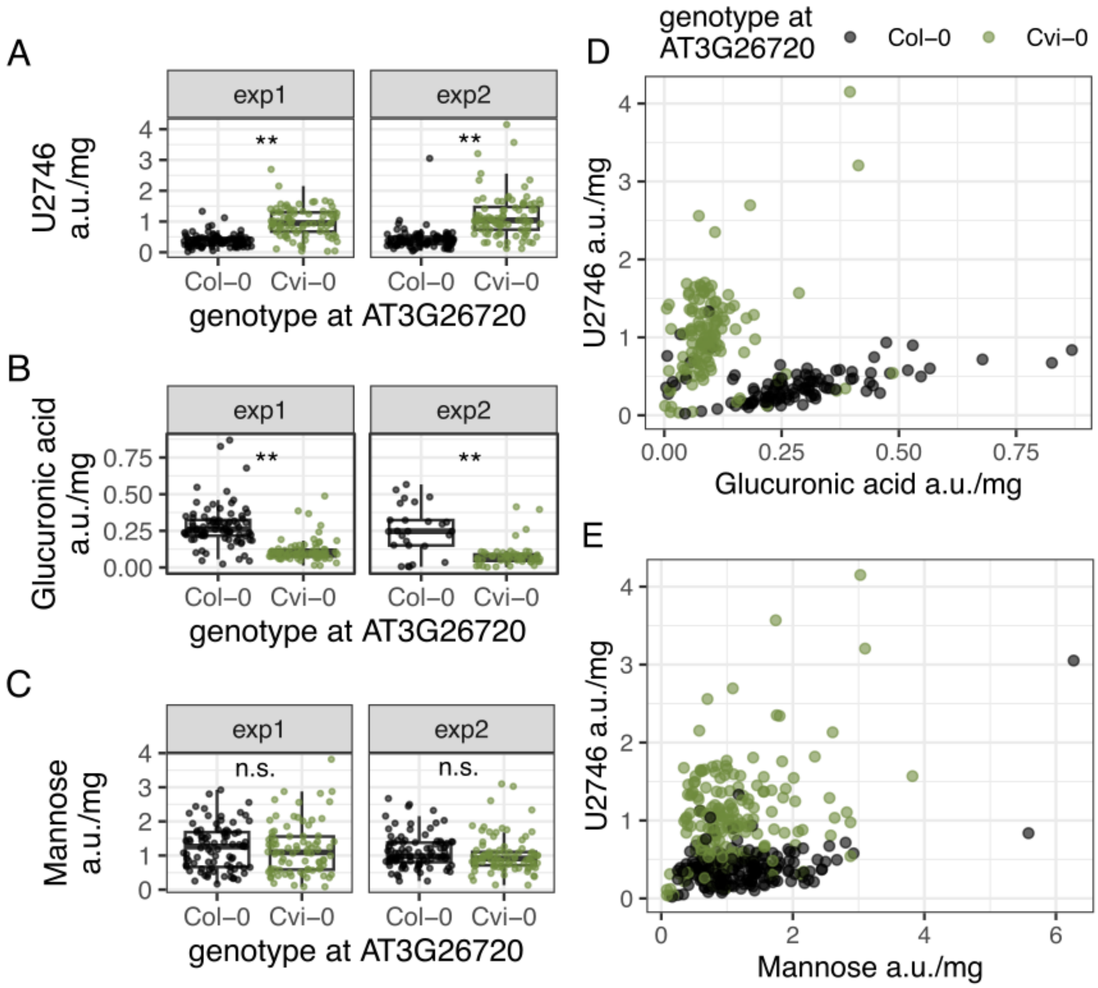

Fig. S7. U2746, glucuronic acid and mannose in the RIL population.
Plants in the RIL population were divided by their genotype at marker 09748 (the closest marker to GH38cv) and their normalized concentration for U2746, glucuronic acid and mannose compared for the concentration of U2746 (A) glucuronic acid (B) and mannose (C). Two asterisks indicate significant differences between the lines (one way ANOVA, $p<0.01$). n.s. stands for not significant. (D) Comparison between levels of U2746 and glucuronic acid in each RIL (E) Comparison between levels of U2746 and mannose in each RIL.

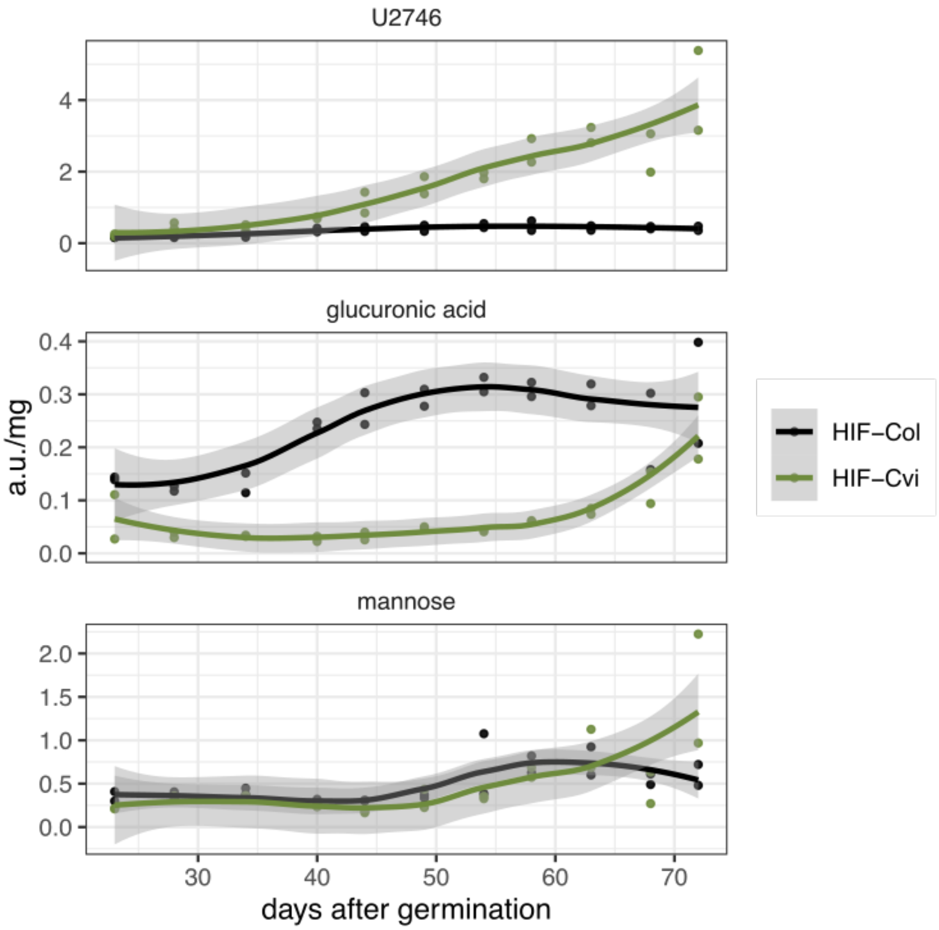

Fig. S8. Developmental time course of U2746

U2746, glucuronic acid and mannose concentration in HIF-Col and HIF-Cvi from day 23 after planting to senescence. Points represent individual leaf samples. Lines are a loess fit per genotype, and the shadowing indicates the 95% confidence level interval for its predictions.

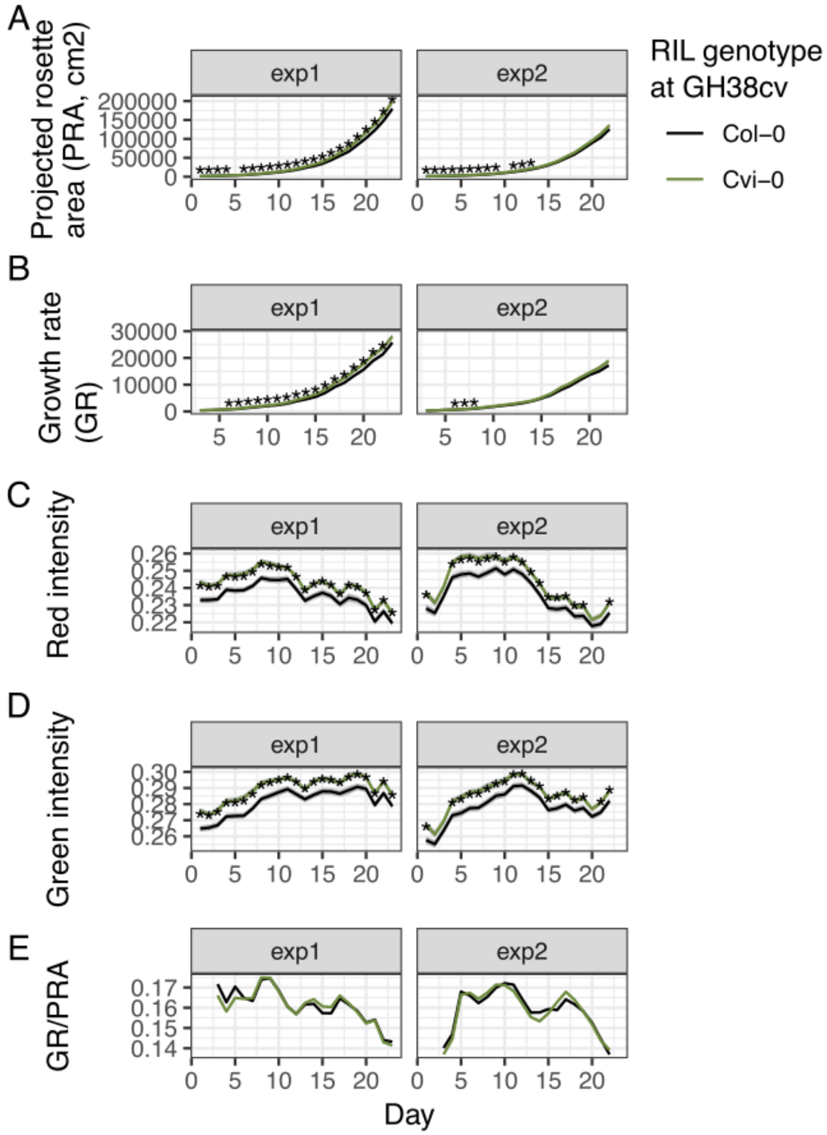

Fig. S9. Characteristics of RILs grouped by their GH38cv allele.
RILs grouped according to their genotype at marker 09748 (the closest marker to GH38cv) were compared for rosette area (A), growth rate (B), red intensity (C) green intensity (D) and growth rate by rosette area (E) using plant images taken during the two experiments for QTL detection in the Phenoscope platform. Asterisks indicate significant differences between RILs with Col-0 or Cvi-0 alleles at 09748 (t-test, $p<0.05$).

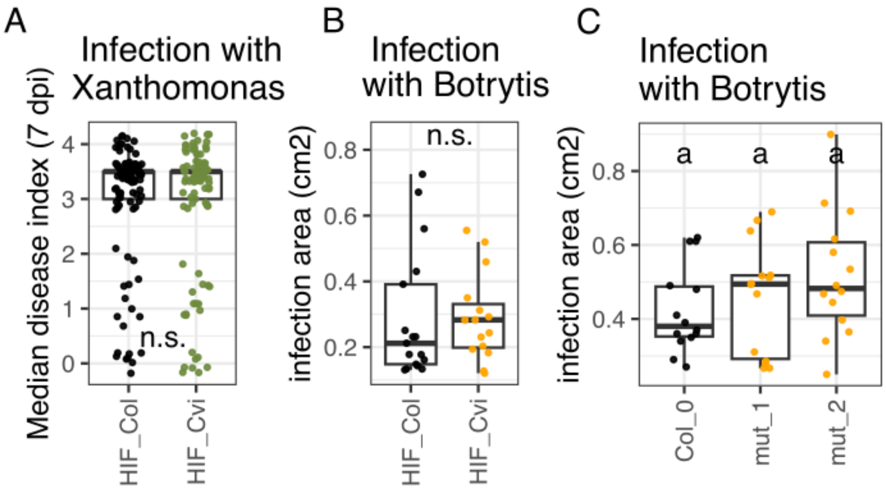

Fig. S10. Effect of GH38cv allele in biotic stress responses.
(A) Virulence assessment taken 7 days post infection in three consecutive experiments. (B and C) Bacterial population density from leaves inoculated with Xanthomonas in the HIF lines segregating for GH38 (B), or in the T-DNA insertion lines for GH38 (C). In (A) and (B) n.s. stands for not significant differences (one-way ANOVA, p>0.05). In (C) different letters indicate significant differences between lines (one-way ANOVA, Tukey HSD test, p<0.05).

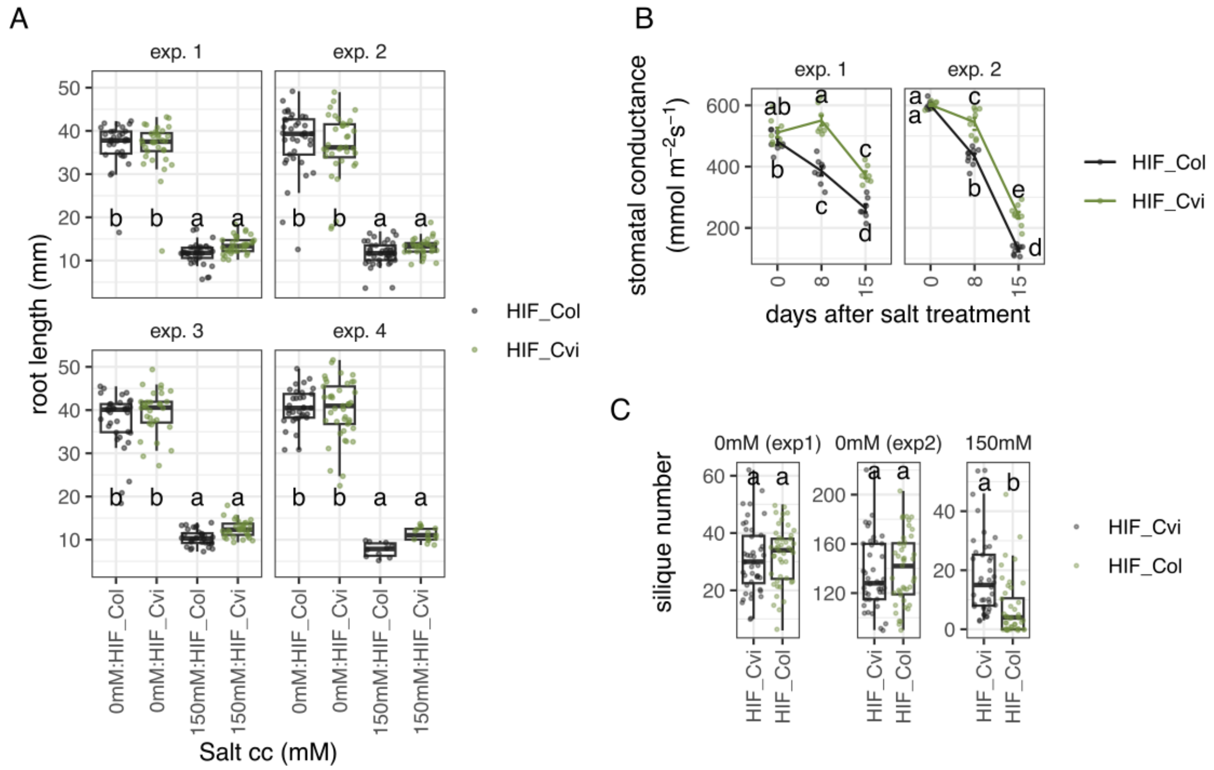

Fig. S11. Effect of mutations in GH38cv in responses to high salinity.
(A) Root length 5 days after transfer to control or salt medium (150mM) in the HIF lines with contrasting alleles at GH38cv. Data from 4 independent experiments are shown separated. Different letters indicate significant differences between lines and conditions (two-way ANOVA, Tukey HSD test, $p<0.05$). (B) Stomatal conductance in plants treated with salted water. Treatment starts at day 0. Two independent experiments are shown. Different letters indicate significant differences between lines and conditions (two-way ANOVA, Tukey HSD test, $p<0.05$). (C) Silique number in plants treated with salted or non-salted water. Two independent experiments in two different growth chambers under control conditions are shown. Different letters indicate significant differences between lines and conditions (two-way ANOVA, Tukey HSD test, $p<0.05$).

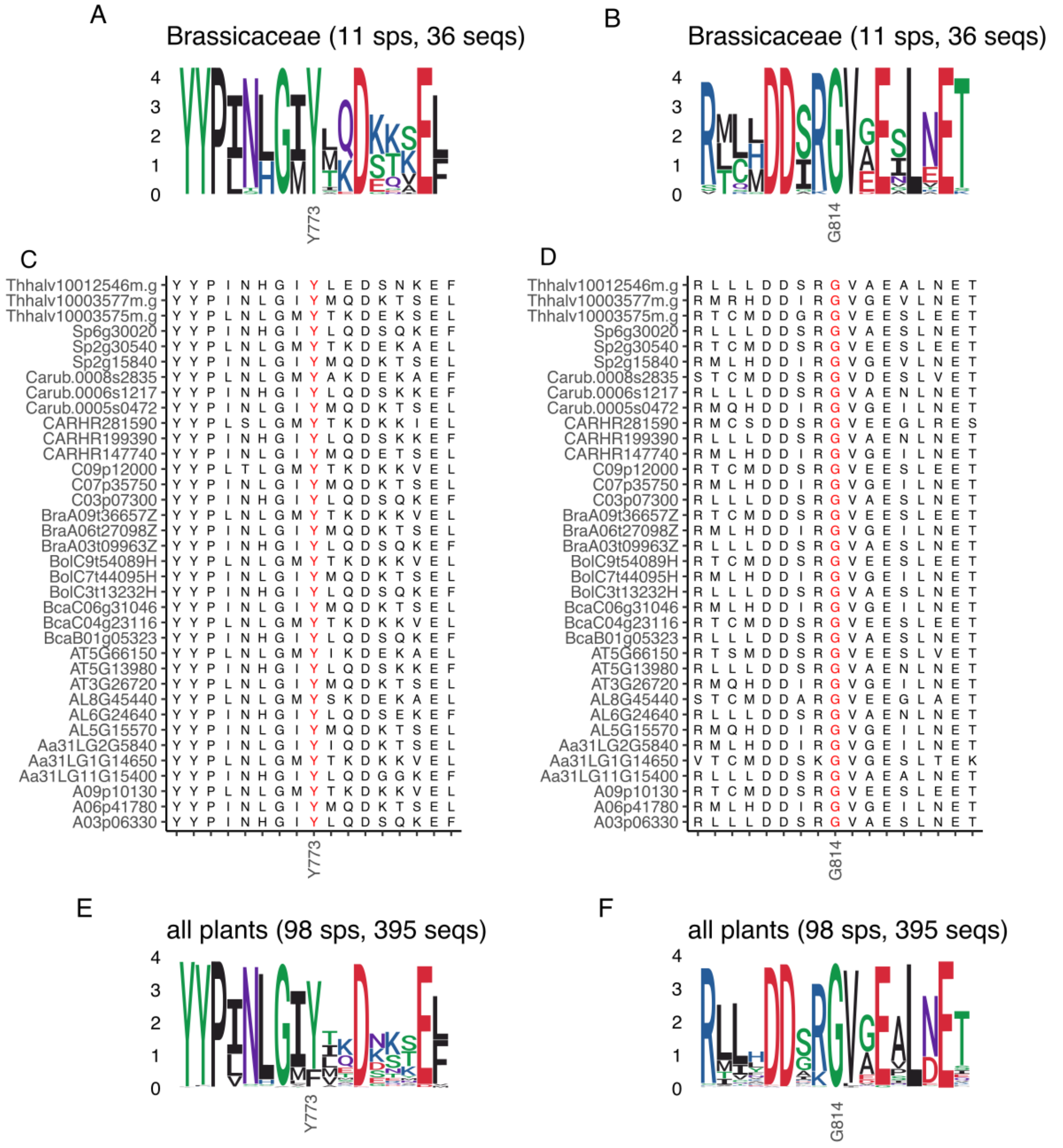

Fig. S12. Allele diversity at positions Y773H and G814D.
(A) and (B) Sequence logo across 36 orthologous sequences from 11 Brassicaceae species for positions Y773H (A) and G814D (B). (c) and (D) are the corresponding alignments represented in (A) and (B). (E) and (F) Sequence logo across 395 orthologous sequences from 98 plant species for positions Y773H (E) and G814D (F) showing higher conservation for the G814D mutation.

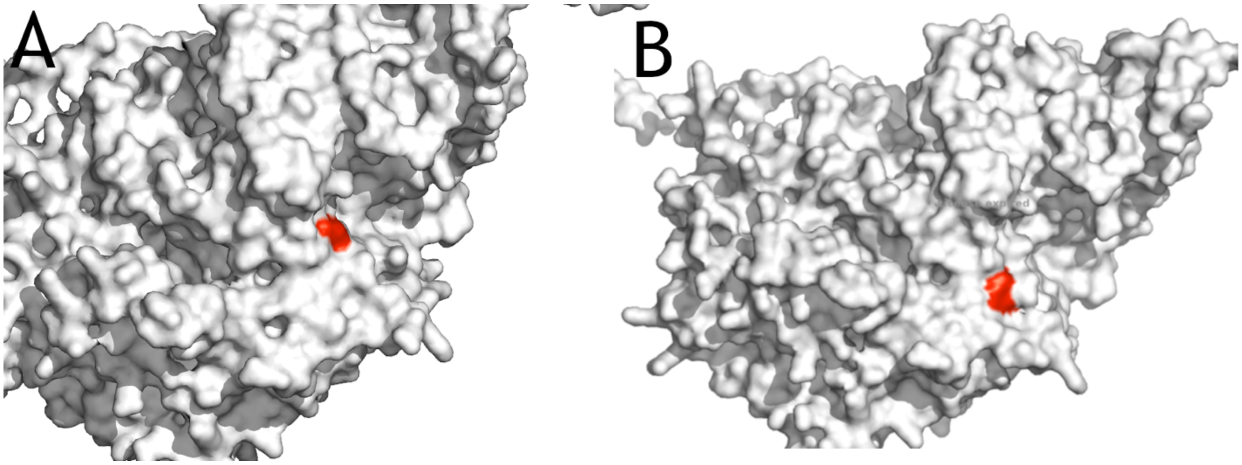

Fig. S13. GH38cv protein model and location of mutations
(A) Protein models obtained from AlphaFold2 for AT3G26720 (A), or for the same protein where we introduced the G814D change (B). Residue 814 is colored in red and seems to be facing a cavity in (A) but facing outside in (B).

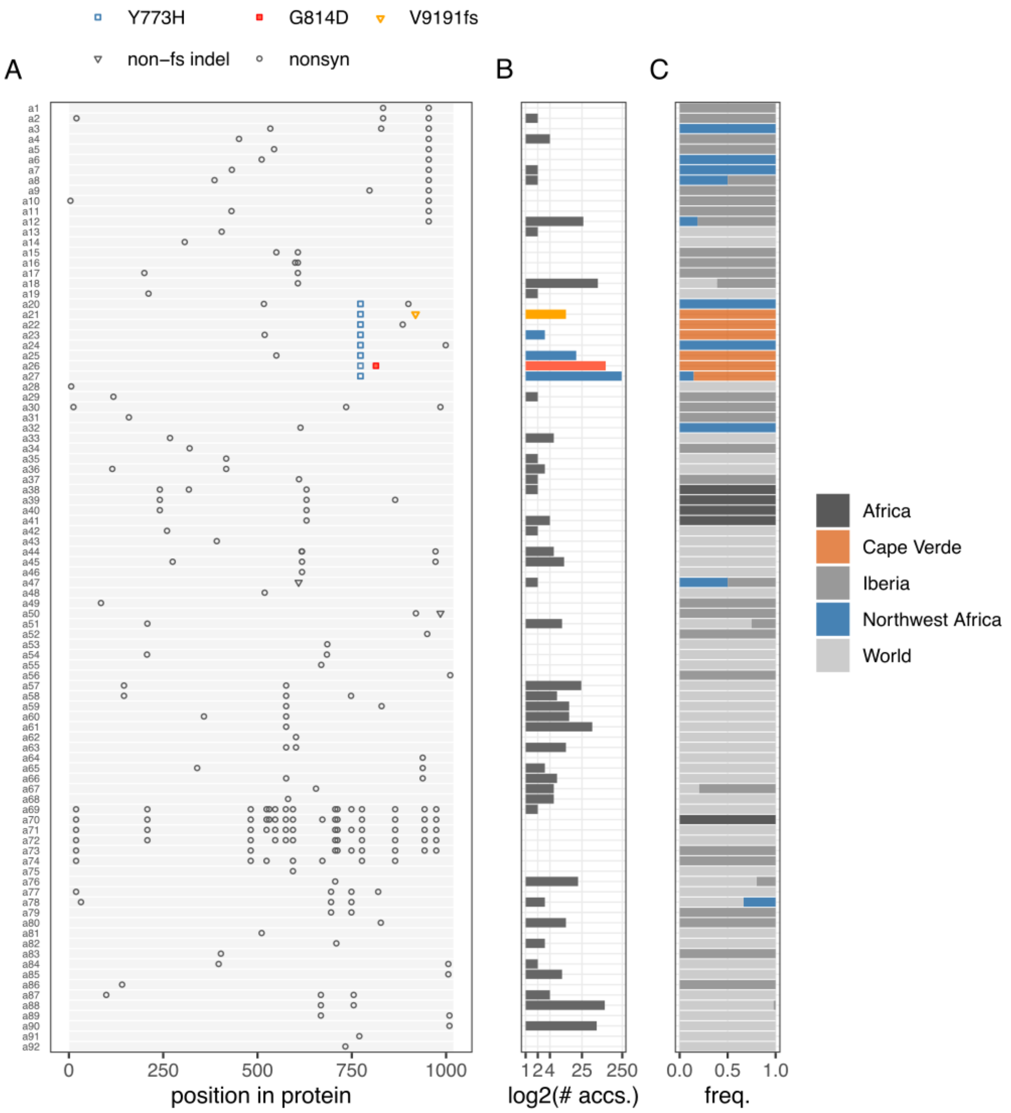

Fig. S14. Allelic diversity at the GH83cv protein in Arabidopsis.
Ninety-two GH38cv alleles were reconstructed using homozygous variants from 1607 re-sequenced Arabidopsis accessions. (A) is a graphical representation of each allele and their mutations with respect to the Col-0 reference. Dark open circles represent nonsynonymous SNPs and dark open triangles represent non frameshift indels. Blue open squares and red filled squares represent the Y773H and G814D mutation found in Cvi-0, respectively. The yellow open triangle represents the V919fs frameshift deletion found in the island of Fogo. (B) represents the number of accessions carrying each allele in log2 scale. Bars from alleles present in the Cape Verde Islands are colored in red if they contain the G814D mutation, in yellow if they contain the V919fs deletion, or in blue if they not carry any of these mutations. (C) represents the frequency of phylogenetic groups for each allele. Note that alleles carrying the G814D or the V919fs mutations are only present in the Cape Verde Islands, suggesting that these mutations originated there.

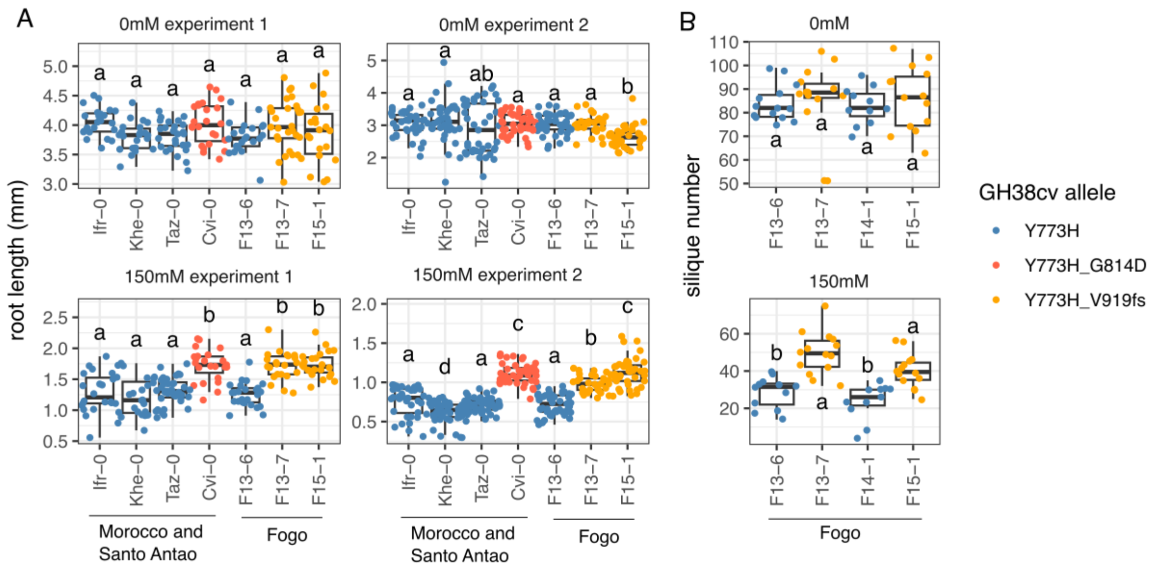

Figure S15. Root length and silique number in accessions from Morocco and Cape Verde Islands under salt treatment

Accessions from Morocco (Ifr-0, Khe-0 and Taz-0) and the Cape Verde islands of Santo Antão (Cvi-0) and Fogo (F13-6, F13-7, F14-1 and F15-1) were phenotyped for root length (A) and silique number (B). Colors represent the different alleles of GH38cv in each accession. (A) shows data from two independent experiments. Different letters indicate significant differences between accessions (two-way ANOVA, Tukey HSD test, $p<0.05$).

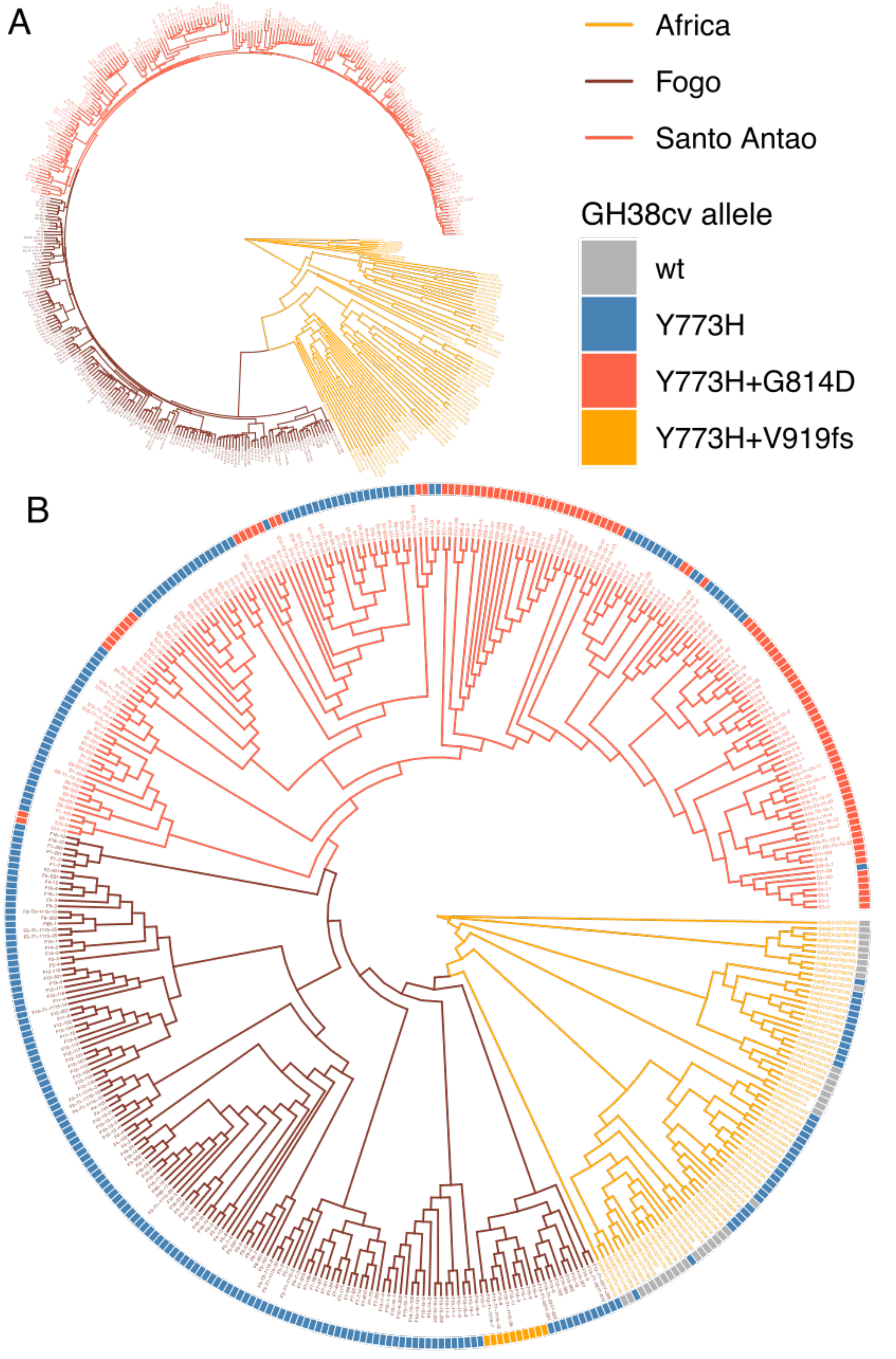

Figure S16. Distribution of GH38cv alleles among African and Cape Verde accessions
Neighbor-joining tree from 6374 fourfold degenerate SNPs segregating among 413 accessions from Africa and the Cape Verde islands of Santo Antão and Fogo. Accessions are colored by the provenance in the passport data. Trees in (A) and (B) are similar. Tree in (A) shows branch lengths. Tree in (B) shows GH38cv allele after the name of each accession.

Table S1. Metabolite concentration in RILs. Comma separated values table with the concentration of each metabolite in each RIL. Information of the Phenoscope experiment, injection into the GC-MS machine, and fresh weight of the material are provided.

Table S2. List of QTLs. Comma separated values table with the list of QTLs identified in the RILs, including metabolite, chromosome, closest marker, positions, LOD score, and effect.

Table S3. Re-sequenced accessions genotyped for GH38. Tab separated values table with all Arabidopsis accessions genotyped for GH38cv with short reads data. The table includes the name of the accession, study where it appeared, region of the world, latitude, longitude and genotype for Y773H, G814D and V919fs, where 0_0 stands for homozygous reference allele (Col-0), 1_1 stands for homozygous alternative allele, and 0_1 stands for heterozygous.

Table S4. Primers used for fine mapping. Excel table with all primers used for fine mapping the U2746 QTL, including information on the expected sizes in Col-0 and Cvi-0.
